# Advances in the Therapeutic Landscape of Hepatocellular Carcinoma: Current Strategies and Future Perspectives

**DOI:** 10.3390/cancers18040609

**Published:** 2026-02-12

**Authors:** Asahiro Morishita, Kyoko Oura, Hiroki Tai, Rie Yano, Mai Nakahara, Tomoko Tadokoro, Koji Fujita, Shima Mimura, Joji Tani, Miwa Tatsuta, Takashi Himoto, Hideki Kobara

**Affiliations:** 1 Department of Gastroenterology and Neurology, Faculty of Medicine, Kagawa University, 1750-1 Ikenobe Miki-cho Kita-gun, Kagawa 761-0793, Japan; morishita.asahiro@kagawa-u.ac.jp (A.M.); tai.hiroki@kagawa-u.ac.jp (H.T.); yano.rie@kagawa-u.ac.jp (R.Y.); nakahara.mai@kagawa-u.ac.jp (M.N.); tadokoro.tomoko@kagawa-u.ac.jp (T.T.); fujita.koji@kagawa-u.ac.jp (K.F.); mimura.shima@kagawa-u.ac.jp (S.M.); tani.joji.kb@kagawa-u.ac.jp (J.T.); kobara.hideki@kagawa-u.ac.jp (H.K.); 2 Department of Gastroenterology KKR Takamatsu Hospital, Tenjinmae 4-18, Takamatsu 760-0018, Kagawa, Japan; tatsuta@kkr-ta-hp.gr.jp; 3 Department of Medical Technology, Kagawa Prefectural University of Health Sciences, Hara 281-1 Mure-cho, Takamatsu 761-0123, Japan; himoto@kagawa-puhs.ac.jp

**Keywords:** hepatocellular carcinoma, cirrhosis, surveillance, Barcelona Clinic Liver Cancer, transarterial chemoembolization, radioembolization, stereotactic body radiotherapy, immune checkpoint inhibitor, tyrosine kinase inhibitor, biomarkers

## Abstract

Hepatocellular carcinoma (HCC) is the most common primary liver cancer and a major cause of cancer mortality worldwide. Because HCC usually arises in cirrhotic livers, prognosis is shaped by the dual threats of tumor progression and hepatic decompensation, requiring treatment decisions that balance anticancer efficacy with preservation of liver function, portal hypertension control, and quality of life. In recent years, management has shifted from a predominantly locoregional approach to an integrated continuum that spans curative resection, ablation, and transplantation; refined transarterial and radiotherapy techniques; and modern systemic therapy dominated by immunotherapy-based combinations. These advances have improved response rates, enabled downstaging and conversion in selected patients, and expanded opportunities for sequential and multimodal treatment. However, challenges persist, including therapeutic decision-making in patients with Child–Pugh B liver function, lack of robust predictive biomarkers, and resistance after initial response. Emerging tools—liquid biopsy, radiomics, AI-assisted imaging, and microbiome modulation—may support future precision strategies and optimized treatment allocation. In this review, we summarize current evidence guiding staging and therapy selection, outline practical considerations across curative, locoregional, and systemic modalities, and discuss evolving biomarkers and next-generation immunotherapy as key steps toward more personalized, durable, and equitable global HCC care.

## Simple Summary

Hepatocellular carcinoma (HCC) arises mostly in chronically diseased livers, so clinicians must manage both an aggressive cancer and a fragile organ simultaneously. This review explains how modern HCC care has evolved into an integrated continuum: from prevention and surveillance to curative options such as resection, ablation, and transplantation, to refined locoregional therapy and immunotherapy-based systemic regimens. We highlight how treatment decisions are tailored according to tumor stage, liver function, portal hypertension, and frailty, and why preserving hepatic reserve is crucial to allow multiple lines of therapy. We also summarize emerging tools such as biomarkers, liquid biopsy, radiomics, and microbiome research that may support more precise treatment selection. Finally, we discuss special populations, safety considerations, and future strategies that combine innovative and traditional approaches to improve survival and quality of life for patients with HCC worldwide. This review aims to guide practical clinical decision-making.

## 1. Introduction

Hepatocellular carcinoma is the most common primary cancer of the liver and usually develops on a background of chronic inflammation and fibrosis [1]. The clinical management of HCC is uniquely constrained by the biology of the host organ [2]. In many patients, the liver is simultaneously the site of malignancy and a failing organ; consequently, the “therapeutic window” is defined not only by tumor stage but also by hepatic reserve, portal hypertension, and systemic frailty [3]. Even when a therapy is oncologically effective, treatment may be discontinued because of worsening jaundice, ascites, encephalopathy, or variceal bleeding [4]. Conversely, aggressive tumor control can stabilize or improve portal flow and liver biochemistry in selected cases, particularly when vascular invasion is reversed [5]. These competing risks mean that clinical decisions must be individualized, dynamic, and iterative over the disease course [6].

Historically, curative-intent modalities—surgical resection, local ablation, and liver transplantation—have provided the best outcomes but are feasible only for a minority of patients at diagnosis [7]. Surveillance programs aim to detect HCC earlier, yet implementation varies across regions and health systems, and many patients continue to present with intermediate or advanced disease [8]. For intermediate-stage tumors, transarterial chemoembolization (TACE) has been widely used for decades [9]. Its role is evolving as technique improves and as systemic therapy becomes more effective and can be introduced earlier for patients in whom repeated embolization would compromise liver function [10]. For advanced disease, the systemic landscape has transformed [11]. Multikinase inhibitors (TKIs) were once the primary systemic option [12]. Today, immune checkpoint inhibitor (ICI)-based combinations and other targeted regimens have expanded first-line choices and created multiple later-line options [13]. These changes are clinically meaningful because higher response rates can translate into symptom relief, prolonged survival, and opportunities for downstaging or conversion to curative-intent strategies in highly selected patients [14].

Despite progress, there are persistent challenges [15]. HCC is biologically heterogeneous, influenced by etiology (viral hepatitis, alcohol, metabolic dysfunction), genetic alterations, and immune microenvironment states [16]. Many patients treated in routine practice have characteristics excluded from pivotal trials, such as Child–Pugh B liver function, extensive portal vein thrombosis with borderline hepatic reserve, or significant cardiovascular and renal comorbidity [17]. Additionally, the optimal sequencing of HCC therapies and the best way to integrate locoregional and systemic approaches are still being defined [18]. The field therefore requires both refined clinical algorithms that prioritize preservation of liver function and translational frameworks that identify which biological contexts are most likely to benefit from specific HCC therapies [19,20,21,22,23,24].

Regional differences in etiologic drivers and health-system resources shape HCC pathways. HBV-related HCC remains predominant in many Asia-Pacific and sub-Saharan African settings, whereas metabolic dysfunction-associated steatotic liver disease (MASLD) and alcohol-related HCC are rising in Western regions. Access to transplantation, locoregional technologies (e.g., TARE, SBRT), and newer systemic agents also varies, and pivotal trial populations may not fully represent local practice. Consequently, guidelines and real-world sequencing differ across regions, underscoring the need to interpret evidence in its geographic and etiologic context.

This review provides a clinically grounded synthesis of the contemporary therapeutic landscape of HCC. We outline evolving epidemiology and etiologic drivers of HCC that shape prevention and surveillance priorities. We then summarize staging systems and practical treatment allocation with an emphasis on liver function. We review curative-intent strategies and locoregional therapies of HCC, followed by systemic regimens and real-world sequencing considerations. Finally, we discuss biomarkers, response assessment, special populations, and future directions aimed at further improving outcomes while maintaining safety and equity.

## 2. Epidemiology, Etiology, and Risk Stratification

### 2.1. Global Burden and Evolving Etiologic Patterns

The global burden of HCC remains substantial, with pronounced geographic variation [25]. Regions with historically high HBV prevalence have carried disproportionate incidence and mortality for decades, while HCV has been a major driver in Japan, parts of Europe, and North America [26]. Population-level trends are now changing [27]. HBV vaccination programs, mother-to-child transmission prevention, and broader access to potent antiviral therapy have reduced new infections and improved long-term outcomes in many settings [28]. HCV elimination efforts and wide deployment of direct-acting antivirals have decreased the pool of individuals with ongoing viremia, though the residual risk of HCC persists in patients with advanced fibrosis [29]. In parallel, the prevalence of obesity, type 2 diabetes, and metabolic dysfunction-associated steatotic liver disease has risen rapidly worldwide [30]. These metabolic drivers are reshaping the patient phenotype, increasing the proportion of older patients with significant cardiovascular risk, chronic kidney disease, and frailty [31].

The shift toward metabolic and mixed-etiology HCC has practical implications [32]. Patients with metabolic liver disease may have more indolent fibrosis progression but can develop HCC in the absence of cirrhosis, complicating the identification of at-risk populations for surveillance [33]. In addition, steatosis and obesity can impair ultrasound performance, and cardiometabolic comorbidities influence treatment tolerance and perioperative risk [34]. As a result, the impact of therapeutic advances may depend as much on effective risk stratification and early detection as on improvements in systemic therapy [35].

### 2.2. Mechanistic Themes Across Etiologies

HBV-related carcinogenesis reflects both indirect and direct mechanisms [36]. Chronic inflammation promotes fibrosis and cirrhosis, while viral integration and viral protein effects can contribute to oncogenesis even without advanced fibrosis [37]. Antiviral therapy that suppresses HBV replication reduces inflammatory activity and lowers HCC risk, but does not eliminate it; surveillance therefore remains essential in high-risk groups, including those with cirrhosis and those with additional risk factors such as older age and family history [38].

HCV-associated HCC is closely linked to fibrosis progression and cirrhosis [39]. Viral eradication reduces necroinflammation and can improve hepatic reserve, yet patients with established advanced fibrosis remain at risk and require continued surveillance [40]. Importantly, post-eradication patients may live long enough for competing comorbidities to influence outcomes; thus, integrated management of metabolic risk and alcohol use becomes increasingly relevant [41].

Alcohol-associated liver disease contributes to HCC through toxic and inflammatory mechanisms, oxidative stress, and iron-related injury, and is often accompanied by malnutrition, sarcopenia, and psychosocial barriers to longitudinal care [42]. Sustained abstinence can improve liver function and reduce complications, potentially expanding treatment options [43]. Therefore, effective HCC care in this population often requires coordinated hepatology, addiction medicine, nutrition, and social support [44].

Metabolic dysfunction-associated steatohepatitis (MASH) contributes through insulin resistance, lipotoxicity, mitochondrial dysfunction, altered bile acid signaling, dysbiosis, and chronic low-grade inflammation [45]. The carcinogenic field effect may develop even with less advanced fibrosis in some patients, likely influenced by genetic susceptibility, adipose tissue inflammation, and immune dysregulation [46]. These mechanistic themes suggest that future preventive and therapeutic strategies may include not only weight reduction and diabetes control but also targeted modulation of metabolic and inflammatory pathways [47].

### 2.3. Surveillance and Risk Stratification in Practice

Surveillance aims to identify HCC at a stage amenable to curative therapy [48]. The most commonly used approach is periodic ultrasound with or without serum tumor markers [49]. In practice, ultrasound sensitivity is reduced in obese individuals, in those with steatosis, and when nodularity is pronounced [50]. Cross-sectional imaging may be used when ultrasound is consistently inadequate or when biomarkers rise without a clear lesion [51]. Effective surveillance requires more than test performance: patient adherence, reminder systems, timely diagnostic workup, and access to definitive therapy all determine real-world benefit [52].

Because the population at risk is large—especially in metabolic liver disease—risk stratification is increasingly important [53]. Fibrosis stage is the strongest predictor across etiologies, but additional factors modify risk, including age, sex, diabetes control, ongoing alcohol intake, viral suppression status, and family history [54]. In clinical settings, practical risk stratification tools must be simple enough for broad implementation while retaining meaningful discrimination [55]. A realistic strategy may involve tiered risk assessment: identifying advanced fibrosis and cirrhosis for routine surveillance, and developing refined models for non-cirrhotic metabolic disease to prioritize surveillance intensity [56].

## 3. Staging Systems and Treatment Allocation

### 3.1. Principles: Tumor Burden, Liver Function, and Performance Status

**Figure 1:**
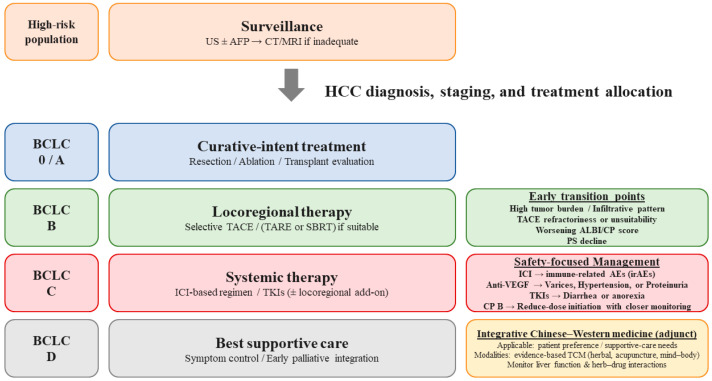
Integrated treatment algorithm for hepatocellular carcinoma. Patients at risk should undergo regular surveillance using ultrasound with or without AFP, with CT/MRI reserved for inconclusive cases. Following diagnosis, treatment strategy is primarily allocated according to BCLC stage, liver function (Child–Pugh and ALBI grade), and performance status. For BCLC 0/A, curative options including surgical resection, ablation, or transplant evaluation are recommended. For BCLC B, locoregional therapy such as selective TACE is considered first-line, while TARE or SBRT may be alternatives where available; evidence-informed integrative supportive approaches (e.g., selected standardized traditional Chinese medicine adjuncts) may be considered as optional supportive care in appropriate patients, with careful attention to hepatic safety and herb–drug interactions. For BCLC C, systemic therapy with immune checkpoint inhibitors and/or tyrosine kinase inhibitors is recommended, and locoregional therapy may be added in selected cases. For BCLC D, supportive and palliative care is indicated. Early transition from locoregional to systemic therapy should be considered in cases of high tumor burden, infiltrative pattern, TACE refractoriness, worsening liver function after local therapy, macrovascular invasion/extrahepatic spread, or decline in performance status. Treatment safety is particularly important in cirrhotic patients; immune-related adverse events (irAEs) must be monitored during ICI therapy, variceal screening is necessary when using anti-VEGF agents, and early supportive management is crucial for TKI-related toxicities. Abbreviations: US, ultrasonography; AFP, α-fetoprotein; CT, computed tomography; MRI, magnetic resonance imaging; HCC, hepatocellular carcinoma; BCLC, Barcelona Clinic Liver Cancer; TACE, transarterial chemoembolization; TARE, transarterial radioembolization; SBRT, stereotactic body radiotherapy; ICI, immune checkpoint inhibitor; TKI, tyrosine kinase inhibitor; ALBI, albumin–bilirubin; CP, Child–Pugh; irAE, immune-related adverse events; VEGF, vascular endothelial growth factor.

Treatment allocation in HCC integrates tumor stage, hepatic reserve, and general condition [3]. Figure 1 shows the integrated treatment algorithm for hepatocellular carcinoma. Staging systems differ by region and purpose, but frameworks that link stage to recommended therapy remain central to clinical decision-making [57]. The Barcelona Clinic Liver Cancer (BCLC) system is widely used because it integrates tumor size and number, vascular invasion or extrahepatic spread, performance status, and liver function to guide therapy [58]. However, real-world decision-making frequently requires nuance beyond fixed categories [59]. Tumor distribution, anatomical considerations, portal hypertension severity, and patient preferences can justify deviations from stage-based recommendations [60].

### 3.2. Assessing Hepatic Reserve and Portal Hypertension

Hepatic reserve is commonly assessed using the Child–Pugh class, which combines bilirubin, albumin, prothrombin time, ascites, and encephalopathy [61]. While clinically intuitive, it includes subjective elements and compresses heterogeneous risk into broad categories [62]. The albumin–bilirubin (ALBI) score provides a continuous laboratory-based measure that can help refine risk within Child–Pugh A [63]. Portal hypertension has independent prognostic and safety implications; it increases the risk of postoperative decompensation and impacts the tolerability of therapies that affect vascular integrity [64]. Clinically, portal hypertension is inferred from platelet count, splenomegaly, varices, and ascites history, and may be directly measured in selected surgical candidates [65].

A practical allocation approach therefore begins by defining whether the patient is compensated or decompensated, whether portal hypertension is clinically significant, and whether a meaningful buffer exists for treatment-related stress [66]. Because hepatic reserve can decline quickly after locoregional therapy, infection, or bleeding, treatment decisions should be revisited at each evaluation rather than assumed to be stable [67].

### 3.3. Treatment Intent and Dynamic Reassessment

Curative intent is pursued when complete tumor eradication is feasible through resection, ablation, or transplantation [68]. In patients outside curative criteria, the goal shifts to durable tumor control and maintenance of liver function [69]. Importantly, the goal can change over time [70]. A patient receiving locoregional therapy may be downstaged and become eligible for liver transplantation [71]. A patient responding to systemic therapy may become a candidate for resection, ablation, or selective radiation to consolidate response [72]. Conversely, a patient initially eligible for repeated TACE may become unsuitable due to declining liver function [73]. Dynamic reassessment—based on tumor response, toxicity, and hepatic reserve—is therefore a core principle of modern HCC management [74].

### 3.4. Practical Assessment of Fitness and Competing Risks

Performance status is a core determinant of treatment eligibility, but in cirrhosis, it often reflects a mixture of cancer symptoms, sarcopenia, anemia, and hepatic encephalopathy rather than cancer alone [75]. Comprehensive geriatric assessment is not feasible in all patients; however, pragmatic measures such as gait speed, grip strength, nutritional screening, and CT-derived sarcopenia can help estimate physiologic reserve and anticipate toxicity [76]. Cardiovascular disease, chronic kidney disease, and diabetes influence both surgical risk and the safety of anti-angiogenic agents and immunotherapy [77]. Renal function and baseline proteinuria are especially relevant when considering VEGF pathway inhibitors, while baseline autoimmune disease and prior organ transplantation are central when considering immune checkpoint blockade [78]. Key practical indicators integrating liver function, portal hypertension, and frailty are summarized in Table 1 [79].

## 4. Curative-Intent Therapy and Locoregional Modalities

Treatment for hepatocellular carcinoma consists of curative therapies, locoregional therapies, and systemic therapies. An overview is summarized in Table 2, which complements the treatment algorithm. Landmark randomized trials and key survival estimates across major modalities are compiled in Table 3.

### 4.1. Surgical Resection

Resection offers a potential cure for selected patients with preserved liver function and resectable disease [80]. Traditional selection emphasizes solitary tumors, limited tumor size, absence of macrovascular invasion, and no clinically significant portal hypertension, though selected patients outside these boundaries may still benefit in expert centers [81]. Advances in imaging, perioperative care, and minimally invasive techniques have expanded eligibility and improved outcomes. Nevertheless, recurrence after resection is common because the cirrhotic liver remains prone to de novo tumor formation and because microscopic vascular invasion may already be present [82].

Optimization strategies aim to reduce perioperative risk and preserve long-term hepatic reserve. Preoperative evaluation includes volumetry, assessment of portal hypertension, cardiopulmonary fitness, and frailty. Nutritional support and sarcopenia assessment are increasingly recognized as important, as muscle loss predicts postoperative complications and poorer survival in HCC patients [83]. Post-resection management focuses on surveillance and control of underlying liver disease [84]. The potential role of adjuvant therapy is evolving, with an emphasis on identifying patients at highest risk for early recurrence based on pathology and imaging features [85].

### 4.2. Local Ablation: Radiofrequency and Microwave Techniques

Percutaneous ablation is a curative-intent option for small lesions, particularly when surgery is contraindicated or when a minimally invasive approach is preferred [86]. Radiofrequency ablation (RFA) and microwave ablation (MWA) are widely used; MWA can generate larger ablation zones and may be less susceptible to heat-sink effects near vessels [87]. Outcomes depend on lesion size, location, and the ability to achieve an adequate margin [88]. Tumors adjacent to major bile ducts or bowel structures require caution, and in challenging locations, laparoscopic or combined approaches can improve safety [89].

Ablation is also commonly used as bridging to transplantation or in combination with transarterial therapy for larger tumors [90]. When a complete response is achieved, the risk of intrahepatic recurrence remains, necessitating continued surveillance [91]. Practical considerations include the choice of imaging guidance, strategies to improve margin control, and standardized follow-up imaging intervals to confirm treatment success and detect early recurrence [92].

### 4.3. Liver Transplantation: Comprehensive Cure for Tumor and Liver Disease

Transplantation offers the most comprehensive curative strategy because it removes both tumor and cirrhotic liver [93]. Eligibility is typically based on tumor burden criteria designed to balance post-transplant recurrence risk with organ allocation ethics [94]. In practice, bridging therapies—ablation, TACE, radioembolization, or radiotherapy—are used to control tumor growth during waiting periods. Downstaging aims to reduce tumor burden into acceptable criteria and can be considered for selected patients with favorable biology, often inferred from response stability over time [95].

Transplant selection increasingly incorporates markers of tumor biology, including response to bridging therapy, AFP trends, and imaging characteristics suggestive of aggressive behavior [96]. Post-transplant recurrence remains a major clinical challenge with limited evidence-based management [97]. Treatment often includes systemic therapy and local control when feasible, while carefully managing immunosuppression and drug interactions. Because rejection risk is heightened with immune checkpoint inhibition, transplantation introduces unique constraints on systemic immunotherapy [98].

### 4.4. Transarterial Chemoembolization (TACE): Principles and Contemporary Practice

TACE has long been the standard for intermediate-stage HCC characterized by multifocal disease confined to the liver without vascular invasion or extrahepatic spread, in patients with preserved liver function [99]. The approach exploits the preferential arterial supply of HCC by delivering chemotherapy and embolic particles to tumor-feeding arteries, inducing ischemic necrosis and local cytotoxicity. Conventional TACE uses an emulsion of chemotherapy and iodized oil followed by embolization, whereas drug-eluting bead (DEB)-TACE releases chemotherapy locally over time [100].

Technique and patient selection strongly influence outcomes of HCC treatments. Superselective catheterization can maximize tumor necrosis while minimizing injury to non-tumoral parenchyma [101]. Repetition may be required, but repeated embolization can progressively impair hepatic reserve [102]. Therefore, defining when TACE is no longer beneficial—because of inadequate response, rapid progression, or worsening liver function—is critical [103]. Concepts such as TACE refractoriness and TACE unsuitability emphasize timely transition to systemic therapy rather than repeated embolization that yields diminishing returns and increases decompensation risk [104].

Practical decision-making after an initial TACE session typically involves response assessment, evaluation of liver function changes, and assessment of residual arterialized viable tumor. A strategy of “on-demand” rather than fixed-schedule repetition can reduce overtreatment. In addition, the presence of extensive bilobar disease, infiltrative morphology, or borderline hepatic reserve may prompt early consideration of systemic therapy rather than TACE as first-line [105].

### 4.5. Transarterial Radioembolization (TARE) and Selective Internal Radiation Therapy

Radioembolization with yttrium-90 microspheres delivers internal beta radiation via the hepatic arterial system with relatively limited embolic effect [106]. This feature can be advantageous in patients with portal vein thrombosis, where embolization could precipitate ischemic injury. TARE can provide durable tumor control and is used for intermediate-stage disease, for downstaging, and for selected advanced-stage cases with predominant liver burden [107]. Treatment planning includes angiographic mapping, assessment of extrahepatic arterial flow, and quantification of lung shunt fraction to avoid radiation pneumonitis [108].

Clinical selection often favors TARE for patients with larger tumors, segmental vascular invasion, or those requiring fewer sessions than TACE [109]. Dosimetry is increasingly individualized, and higher absorbed doses to the tumor can improve response while maintaining safety for the remaining liver [110]. TARE can also be used in a “radiation segmentectomy” approach for early-stage lesions in locations where ablation is difficult, providing a local control alternative with a favorable safety profile in experienced centers [111].

### 4.6. External Beam Radiotherapy and Proton Therapy

Modern radiotherapy, including stereotactic body radiotherapy (SBRT) and proton therapy, has expanded the role of radiation in HCC [112]. Improved imaging guidance, motion management, and conformal planning allow delivery of ablative doses while sparing non-tumoral liver [113]. Radiotherapy can be used for local control of lesions not amenable to ablation or transarterial therapy, as a bridge to transplant, or as palliation for painful bone lesions [114]. In selected cases, radiotherapy provides local control of vascular invasion, potentially improving portal flow and liver function.

Radiotherapy requires careful assessment of liver reserve, as baseline hepatic impairment increases the risk of radiation-induced liver disease [115]. Fractionation, target selection, and dose constraints are tailored to the remaining functional liver volume. The integration of radiotherapy with systemic therapy is an active area of clinical development, with particular interest in combining SBRT with immunotherapy to enhance systemic immune responses through immunogenic cell death and antigen release [116].

### 4.7. Hepatic Arterial Infusion Chemotherapy (HAIC) and Regional Chemotherapy Approaches

HAIC delivers chemotherapy directly into the hepatic artery through an implanted catheter-port system or temporary catheterization [117]. It is used in some regions for patients with extensive intrahepatic disease or portal vein invasion. Potential advantages include high intrahepatic drug exposure with reduced systemic toxicity. However, HAIC requires specialized infrastructure, careful catheter maintenance, and management of complications such as infection, thrombosis, or catheter dislodgement [118].

As systemic therapy response rates improve, the comparative role of HAIC is being re-evaluated [119]. In practice, HAIC may remain relevant for patients with predominant liver tumor burden where rapid local control is needed, particularly when systemic options are limited by hepatic reserve or comorbidity [120]. Combination strategies that integrate HAIC with immunotherapy or TKIs are being explored to leverage local cytotoxicity and systemic immune activation [121].

### 4.8. Emerging and Complementary Locoregional Approaches

The locoregional toolbox continues to expand beyond conventional ablation and transarterial therapy [122]. Irreversible electroporation can ablate tumors using nonthermal mechanisms and may be considered near bile ducts or vascular structures where thermal ablation risks injury, though availability and experience vary [123]. High-intensity focused ultrasound and other noninvasive ablation technologies are being explored in selected settings [124]. Endoscopic ultrasound-guided therapies have been reported for lesions in challenging locations, potentially enabling targeted ablation or injection approaches under direct endoscopic guidance [125]. These innovative techniques may enhance treatment options for hepatocellular carcinoma, particularly in challenging cases where traditional methods pose risks to surrounding structures.

Combination locoregional strategies for HCC are also evolving [126]. For example, TACE can be used to reduce tumor size and vascularity prior to ablation to improve margin control. Radioembolization can be used to induce contralateral lobe hypertrophy (“radiation lobectomy”), potentially enabling later resection in patients with insufficient future liver remnant [127]. These approaches highlight the importance of individualized planning and the need to view locoregional therapy not as a single procedure but as a set of modular interventions that can be sequenced to achieve defined goals: local control, downstaging, bridging, or symptom palliation [128]. As the treatment landscape for HCC continues to evolve, integrating innovative therapies with traditional approaches will be crucial for optimizing patient outcomes. The integration of innovative locoregional therapies with systemic treatments is essential for optimizing outcomes in hepatocellular carcinoma, especially in patients with complex clinical profiles.

Beyond procedure-based innovation, supportive modalities are increasingly used to address symptom burden during locoregional therapy. Non-pharmacological approaches such as acupuncture/acupressure may help with fatigue, pain, nausea, and sleep disturbance in selected patients as adjunctive supportive care. When standardized traditional Chinese medicine (TCM) products are used, clinicians should prioritize formulations with quality control, avoid potentially hepatotoxic preparations, and proactively assess herb–drug interactions with TKIs, anti-VEGF agents, and ICIs. Evidence remains heterogeneous; therefore, integrative strategies should be individualized and framed as supportive care within a multidisciplinary plan rather than as a substitute for evidence-based oncologic therapy.

## 5. Systemic Therapies: Current Standards and Practical Sequencing

### 5.1. First-Line Systemic Therapy: Immunotherapy-Based Combinations and Alternatives

Systemic therapy is indicated for advanced-stage disease, for patients with macrovascular invasion or extrahepatic spread, and increasingly for those with intermediate-stage disease that is unsuitable for or refractory to transarterial therapy [129]. The first-line landscape is now dominated by immunotherapy-based combinations. Regimens combining immune checkpoint blockade with VEGF pathway inhibition exploit complementary mechanisms: VEGF inhibition can normalize tumor vasculature, reduce immunosuppressive myeloid cell recruitment, and improve immune cell trafficking, potentially enhancing ICI activity [130]. These combinations have demonstrated improved survival and higher response rates compared with older TKI standards and have become widely adopted in clinical practice [131].

Dual immune checkpoint blockade—combining PD-1 or PD-L1 inhibitors with CTLA-4 inhibition—represents another first-line approach. CTLA-4 blockade can enhance T-cell priming and broaden antitumor immunity, while PD-1/PD-L1 blockade sustains effector function within the tumor microenvironment [132]. In practice, dual ICI regimens may be selected when anti-VEGF therapy is contraindicated, for example, in patients with high bleeding risk or those who cannot undergo appropriate variceal assessment and prophylaxis [133]. However, dual ICI approaches can increase the risk of immune-related adverse events, requiring careful patient selection and monitoring [134].

Multikinase inhibitors remain important first-line options for patients in whom immunotherapy is contraindicated, such as those with active autoimmune disease requiring immunosuppression, a history of severe immune-related toxicity, or selected post-transplant contexts where rejection risk is prohibitive [135]. TKIs target angiogenesis and proliferative signaling pathways, can be administered orally, and have well-established dose-modification strategies [136]. Choosing among first-line options requires a structured assessment of portal hypertension and bleeding risk, autoimmune history, infection risk, cardiovascular status, renal function, and baseline liver reserve [137].

### 5.2. Second-Line and Later-Line Options: Principles Rather than Rigid Algorithms

Multiple agents are available beyond first line, including additional TKIs and anti-angiogenic monoclonal antibodies used in selected biomarker-defined contexts in HCC. Immune checkpoint inhibitors may also be used in later lines, depending on prior therapy exposure and regional approvals [138]. The proliferation of options underscores the importance of sequencing principles rather than rigid algorithms in HCC [139].

A key principle is the preservation of liver function [140]. The ability to receive second- and third-line therapy strongly depends on maintaining hepatic reserve during earlier treatment [141]. Therefore, proactive management of portal hypertension, nutrition, and adverse events is not ancillary but central to extending survival [142]. Another principle is mechanism-informed switching: when progression occurs on a VEGF pathway inhibitor, switching to a TKI with a different target spectrum may provide benefit; when intolerance drives discontinuation, selecting an agent with a different toxicity profile is rational. A third principle is pacing and reassessment: patients with indolent progression and preserved function may benefit from continued therapy beyond radiographic progression in selected contexts, whereas rapid symptomatic progression may require swift switching or integration of local palliation [143].

Practical later-line selection also depends on the pattern of progression [144]. When intrahepatic progression is predominant and liver function is preserved, it is worthwhile to consider localized ablative therapies such as TARE or SBRT in conjunction with continued systemic therapy. Predominant extrahepatic progression may prompt switching to an agent with systemic activity [145]. Tumor markers and symptoms can provide additional clues to biological aggressiveness and urgency.

### 5.3. Systemic Therapy in Intermediate-Stage Disease: Moving Earlier in the Continuum

Intermediate-stage HCC is heterogeneous, ranging from limited multifocal disease well suited to superselective TACE to diffuse bilobar disease with high tumor burden that is unlikely to respond durably to embolization [146]. In the latter group, repeated TACE can compromise liver function without achieving meaningful disease control [147]. As systemic therapy response rates have increased, an “earlier systemic therapy” strategy has gained momentum for patients with high tumor burden, infiltrative morphology, or poor suitability for selective embolization [148].

A practical approach involves an initial attempt at high-quality superselective TACE for appropriate candidates, followed by early response evaluation [104]. If viable tumor persists extensively, if progression occurs quickly, or if liver function declines, transitioning to systemic therapy is often preferable to repeated TACE [149]. This approach aims to preserve hepatic reserve and maintain eligibility for multiple therapy lines [148]. Importantly, the decision is not binary: many patients benefit from combination or sequential strategies, such as systemic therapy induction followed by selective locoregional consolidation in responding lesions [150].

### 5.4. Conversion, Downstaging, and Multidisciplinary Integration

Higher response rates with modern systemic regimens for HCC have created new opportunities for conversion and downstaging strategies [151]. In selected patients with initially unresectable disease, major tumor shrinkage or necrosis can enable surgical resection, ablation, or transplantation consideration after sustained response and careful assessment of tumor biology [152]. Conversion strategies are most feasible when liver function is preserved, when response is deep and durable, and when residual disease becomes anatomically amenable to local control [153].

Because conversion carries risk—including postoperative decompensation and recurrence—HCC patient selection and timing are critical [154]. A multidisciplinary framework is essential to determine whether surgery or local therapy adds meaningful benefit beyond continued systemic therapy [18]. In addition, perioperative planning must consider the pharmacology of anti-angiogenic agents, which can impair wound healing and increase bleeding risk, and immunotherapy, which may influence perioperative inflammation and hepatic injury [155].

### 5.5. Response Assessment and When to Switch Therapy

Response assessment in HCC integrates imaging, biomarkers, symptoms, and liver function [156]. For immunotherapy, delayed responses and atypical response patterns can occur, and radiographic progression may occasionally reflect immune cell infiltration rather than tumor growth [157]. Nevertheless, true hyperprogression and rapid clinical decline can also occur [158]. Therefore, clinical judgment is required. A structured approach for HCC considers the magnitude and rate of radiographic change, new lesion development, symptom trajectory, tumor marker trends, and hepatic reserve [159]. In many cases, continued therapy with close follow-up is reasonable when the patient is clinically stable and progression is minimal [156]. Switching is favored when progression is substantial, when symptoms worsen, when liver function deteriorates due to tumor burden, or when toxicity limits continued therapy.

### 5.6. Managing Portal Vein Thrombosis and High-Risk Bleeding Contexts

Macrovascular invasion, particularly portal vein tumor thrombus, is a major turning point in HCC management [160]. Embolization-based therapies carry a higher risk when portal flow is compromised, yet regional and institutional practices differ. Radioembolization is often considered because its embolic effect is limited. Radiotherapy can also target tumor thrombus to achieve local control and potentially restore flow. Systemic therapy remains central, and rapid initiation may be warranted when tumor thrombus is extensive or when symptoms of portal hypertension are worsening [161].

Bleeding risk is a recurring issue because many patients have esophageal or gastric varices, thrombocytopenia, and coagulopathy [162]. Before initiating anti-angiogenic therapy, clinicians should assess and mitigate variceal bleeding risk through endoscopy when appropriate and manage portal hypertension using beta-blockers or ligation [163]. Anticoagulation decisions in cirrhosis are complex; portal vein thrombosis unrelated to tumor can occur and may require anticoagulation, but the risk-benefit balance must be individualized [164]. In managing hepatocellular carcinoma with portal vein tumor thrombosis, careful consideration of systemic therapy options and potential complications is essential for optimizing patient outcomes.

## 6. Biomarkers, Response Assessment, and the Path Toward Precision Medicine

### 6.1. Serum Biomarkers and Longitudinal Dynamics

AFP remains the most widely used serum biomarker. Its absolute value and trajectory can correlate with tumor burden and treatment response, though sensitivity is limited because a substantial fraction of tumors does not secrete AFP [165]. Other biomarkers, such as des-gamma-carboxy prothrombin and AFP-L3, are used in some regions and can complement AFP [166]. In practice, biomarkers are most useful when tracked longitudinally and interpreted alongside imaging [167]. A declining AFP after therapy can support response even if imaging changes are subtle, while a rising AFP can prompt closer imaging or earlier switching [168].

### 6.2. Imaging-Based Response: Viable Tumor Concepts and Functional Assessment

Because HCC therapies often induce necrosis without dramatic size reduction, response criteria that evaluate viable enhancing tumor are commonly used, especially after locoregional therapy. For systemic therapy, both size and enhancement patterns matter, and the appearance of new lesions is a key driver of progression designation [169]. Advanced imaging techniques such as diffusion-weighted MRI can provide additional information about cellularity and necrosis in HCC [170]. Functional imaging and quantitative methods may add value for early response detection, though standardization remains a challenge [171]. The integration of advanced imaging techniques and serum biomarkers is crucial for accurately assessing treatment response in hepatocellular carcinoma, particularly in the context of evolving therapeutic strategies [156]. Future research should focus on developing multiparametric models that combine imaging and biomarker data to enhance the precision of treatment response assessments in HCC management [156]. This approach aims to refine the understanding of treatment efficacy and optimize patient outcomes in hepatocellular carcinoma management. This integrated approach is essential for improving response assessment accuracy and tailoring treatment strategies to individual patient needs in hepatocellular carcinoma management.

Imaging also provides prognostic features beyond response during the treatment of HCC [172]. Radiographic signs suggestive of microvascular invasion, peritumoral enhancement, and infiltrative morphology correlate with recurrence risk and aggressive behavior [173]. Incorporating these features into treatment selection and surveillance intensity is an emerging practice goal. Incorporating advanced imaging techniques and serum biomarkers into routine clinical practice will enhance the precision of treatment response assessments and improve patient outcomes in hepatocellular carcinoma management [156]. This integrated approach is essential for improving response assessment accuracy and tailoring treatment strategies to individual patient needs in hepatocellular carcinoma management.

### 6.3. Tumor Biology and the Microenvironment: Why Predictive Biomarkers Are Hard

HCC arises through diverse genetic and epigenetic alterations [174]. Common pathways include telomere maintenance, cell-cycle dysregulation, WNT/β-catenin signaling, oxidative stress response, and chromatin remodeling [175]. However, the translation of genomic alterations into actionable treatment selection has been limited [176]. Tissue sampling is not always performed because radiologic diagnosis is often sufficient, and multifocal tumors may be heterogeneous [177]. Furthermore, tumor-intrinsic alterations interact with immune and stromal components, creating microenvironment states that influence immunotherapy responsiveness in HCC [178]. The complexity of the immune microenvironment in HCC underscores the need for robust biomarkers to predict treatment responses, particularly in the context of immunotherapy. Addressing these challenges will be crucial for advancing personalized treatment strategies and improving patient outcomes. The integration of advanced imaging techniques with biomarker analysis could significantly enhance our ability to predict treatment responses and tailor therapies for HCC patients, ultimately improving clinical outcomes.

Nevertheless, several themes inform precision efforts. Immune-inflamed tumors characterized by T-cell infiltration may be more likely to respond to immunotherapy, while immune-excluded tumors with active WNT/β-catenin signaling and dense stromal barriers may be less responsive [179]. Angiogenic signatures and hypoxia-related programs may influence benefit from VEGF pathway inhibition [180]. Myeloid-driven immunosuppression, including tumor-associated macrophages and neutrophils, can limit T-cell activity [181]. These observations support composite biomarker strategies that integrate tumor genomics, transcriptomics, immune profiling, and clinical features [182]. Recent integrative bioinformatic approaches coupled with functional validation have also nominated extracellular matrix regulators such as lumican as candidate antineoplastic and prognostic factors in HCC [183].

### 6.4. Liquid Biopsy and Minimal Residual Disease Monitoring

Liquid biopsy approaches, including circulating tumor DNA (ctDNA), circulating tumor cells, and exosomal RNA, offer a noninvasive route to monitor tumor evolution. In HCC, liquid biopsy is particularly attractive because tumors are often multifocal and tissue may not be available [184]. Potential applications include detection of minimal residual disease after curative therapy, early identification of recurrence, and monitoring of resistance mechanisms during systemic therapy. However, sensitivity can be limited by low shedding, especially in small tumors, and results can be confounded by clonal hematopoiesis and non-tumor DNA sources [185]. Standardization of assays, thresholds, and clinical decision rules is necessary before broad implementation [186]. Emerging technologies, such as artificial intelligence and advanced imaging, hold promise for enhancing the accuracy of liquid biopsy techniques, potentially transforming HCC management through improved biomarker discovery and monitoring [187]. Incorporating liquid biopsy techniques into routine clinical practice could significantly enhance the monitoring of treatment responses and disease progression in hepatocellular carcinoma, ultimately improving patient management and outcomes. The integration of liquid biopsy techniques into clinical practice is expected to enhance the monitoring of treatment responses and disease progression in hepatocellular carcinoma, thus improving patient management and outcomes.

### 6.5. AI, Radiomics, and Digital Pathology as Decision-Support Tools

Artificial intelligence methods can extract quantitative features from imaging that reflect tumor heterogeneity, vascular architecture, and peritumoral environment. Radiomics-based models have been proposed for predicting microvascular invasion, recurrence risk, and treatment response [188]. Digital pathology can quantify immune cell distributions, stromal density, and spatial relationships that are difficult to capture by conventional scoring [189]. These tools may eventually support more consistent staging, improved surveillance quality control, and individualized risk prediction [190].

For clinical adoption, AI tools must demonstrate robust performance across centers, scanners, and populations, and must be integrated into workflows in a way that supports clinicians rather than adds burden [191]. Interpretability and bias assessment are essential, particularly as HCC epidemiology shifts and imaging characteristics change with metabolic disease prevalence [192].

### 6.6. Microbiome, Inflammation, and Systemic Therapy Responsiveness

The gut–liver axis shapes hepatic immunity and has emerged as a plausible modifier of therapy response [193]. Cirrhosis is associated with altered gut permeability, dysbiosis, and systemic inflammation [194]. These factors influence myeloid cell activation and may contribute to immune suppression within the liver [195]. Although clinical translation remains early, an emerging supportive framework is to reduce bacterial translocation and inflammation through diet quality optimization, constipation control, and prudent antibiotic stewardship, and to evaluate targeted probiotics/prebiotics or bile-acid modulation in carefully selected patients within evidence-based pathways. More intensive approaches such as fecal microbiota transplantation should be considered investigational and restricted to research settings. Importantly, microbiome-directed strategies remain adjunctive and unproven as efficacy enhancers in intermediate-stage combination regimens; prospective validation is required before routine integration [196].

### 6.7. Post-Treatment Surveillance and Recurrence Management

Recurrence after curative-intent therapy is common and can be intrahepatic, extrahepatic, or both [197]. A practical surveillance program after resection, ablation, or transplantation includes scheduled cross-sectional imaging and tumor marker assessment with intervals that reflect the highest-risk period during the first two years [198]. When recurrence is detected, the decision framework mirrors primary treatment allocation but must incorporate prior therapies and residual hepatic reserve. For small, isolated intrahepatic recurrences, repeat ablation or limited resection can be considered when feasible [199]. For multifocal recurrence in a compensated liver, transarterial therapy may provide control, while early systemic therapy should be considered when the recurrence pattern suggests aggressive biology or when locoregional options would require repeated procedures with high risk of liver injury. Overall, the evolving landscape of HCC management necessitates a continuous reassessment of treatment strategies to optimize patient outcomes and adapt to emerging evidence in this dynamic field.

## 7. Safety, Adverse Events, and Special Populations

### 7.1. Immune-Related Adverse Events in Patients with Chronic Liver Disease

Immune checkpoint inhibitors can cause immune-related adverse events (irAEs) involving the skin, endocrine organs, gastrointestinal tract, lungs, and liver. In HCC, immune-mediated hepatitis can be difficult to distinguish from disease progression or underlying cirrhosis; baseline liver tests, trajectory, and imaging context are essential for interpretation [200,201]. Management follows general irAE algorithms with early recognition, severity grading, corticosteroids when indicated, and specialist input for steroid-refractory cases, while balancing the risk of infection and hepatic decompensation in cirrhosis [202,203].

### 7.2. VEGF Inhibition and TKI Toxicities: Proactive Mitigation

Anti-angiogenic therapy can cause hypertension, proteinuria, bleeding, impaired wound healing, and thrombosis [204]. In cirrhosis, bleeding risk is strongly influenced by portal hypertension and varices [205]. Therefore, baseline endoscopic evaluation and appropriate prophylaxis (nonselective beta-blockers or variceal ligation as indicated) are important before initiating agents with bleeding risk [206]. TKIs commonly cause hand–foot skin reaction, diarrhea, fatigue, appetite loss, and weight loss [207]. Early supportive care, dose modifications, and patient education can prevent treatment discontinuation and preserve quality of life [208]. Importantly, toxicity management is also a liver-preservation strategy: dehydration from diarrhea or reduced intake can precipitate renal dysfunction and worsen ascites, indirectly reducing eligibility for subsequent therapy [209]. Incorporating a multidisciplinary approach is vital for managing immune-related adverse events, particularly in patients with underlying liver conditions, to optimize therapeutic outcomes and minimize complications. In patients with advanced liver disease, careful monitoring for immune-related adverse events is essential to balance the benefits of immunotherapy with potential complications [210].

### 7.3. Patients with Child–Pugh B and Other Trial-Excluded Groups

Most pivotal systemic trials enrolled patients with Child–Pugh A liver function [211]. In routine practice, many patients have Child–Pugh B liver function, and decisions are more complex [196]. Drug exposure may be higher, toxicity tolerance lower, and the competing risk of liver failure greater [212]. A pragmatic approach involves careful selection based on the degree of decompensation, performance status, and patient goals [213]. Dose reduction, slower escalation, and close monitoring may be appropriate for some agents. For others, best supportive care or palliative locoregional control may be preferred [214]. Multidisciplinary management of ascites, encephalopathy, and portal hypertension can sometimes stabilize hepatic reserve and allow therapy that would otherwise be unsafe [215]. Incorporating a multidisciplinary approach is essential for optimizing treatment outcomes and managing the complexities of hepatocellular carcinoma in patients with compromised liver function. Incorporating these considerations into treatment planning can significantly enhance patient outcomes, particularly in those with advanced liver disease and complex clinical profiles.

### 7.4. Frailty, Sarcopenia, and Patient-Centered Outcomes

Frailty and sarcopenia are common in cirrhosis and worsen outcomes across HCC treatments. Muscle loss reduces treatment tolerance, increases postoperative complications, and is associated with poorer survival [216]. Therefore, assessment of nutrition and function should be part of baseline evaluation and follow-up [217]. Interventions may include individualized dietary counseling with adequate protein intake, late-evening snacks to reduce catabolism, resistance exercise adapted to cirrhosis limitations, and management of contributing factors such as hypogonadism or chronic inflammation when appropriate [218]. Patient-reported outcomes capture fatigue, appetite, pain, and functional impact, providing additional decision support when balancing therapy intensity against quality of life [219].

### 7.5. Health Economics, Access, and Value-Based Sequencing

Drug access and affordability increasingly influence real-world sequencing, particularly as first-line regimens shift toward high-cost combinations, and as multiple later-line options become available. Cost-effectiveness estimates are highly context-dependent (pricing, reimbursement, health-state utilities, and willingness-to-pay thresholds). For example, a published cost-utility analysis in France estimated an incremental cost-utility ratio of approximately €153,000 per QALY gained for atezolizumab plus bevacizumab versus sorafenib (roughly US $169,000 per QALY using contemporary exchange rates) [220]. Health-economic evaluation for integrative supportive approaches remains a research gap and should be embedded into prospective trials and implementation studies, alongside patient-reported outcomes and equity metrics.

## 8. Future Directions

### 8.1. Next-Generation Immunotherapy and Microenvironment Modulation

Beyond PD-1/PD-L1 and CTLA-4 blockade, future immunotherapies aim to improve response depth and overcome resistance [221]. Strategies include bispecific antibodies that redirect T cells to tumor antigens, agonists that enhance costimulatory signaling, and therapies that reprogram immunosuppressive myeloid cells [222]. Modulation of the tumor microenvironment—reducing hypoxia, normalizing vasculature, and altering stromal barriers—may increase immune infiltration and synergize with checkpoint blockade [223]. Because cirrhosis is an immune-altered state, these therapies must be developed with careful attention to hepatic safety [224].

### 8.2. Cellular Therapy, Vaccines, and Locally Delivered Immunomodulation

Adoptive cellular therapies and therapeutic vaccines represent additional avenues [225]. Cellular therapies face challenges in solid tumors, including antigen selection, trafficking, and on-target off-tumor toxicity [226]. Vaccines and oncolytic viruses may be used to prime immunity and convert “cold” tumors to “hot” immune states [227]. Locally delivered immunomodulatory agents—via intratumoral injection or catheter-based approaches—could limit systemic toxicity and leverage the accessibility of the liver for interventional procedures [228].

### 8.3. Precision Prevention and Implementation Science

The exploration of novel treatment strategies, including the integration of next-generation immunotherapies and localized approaches, holds promise for enhancing patient outcomes in hepatocellular carcinoma management. The greatest population-level benefit may come from prevention and early detection [229]. HBV vaccination and antiviral therapy, HCV elimination, alcohol harm reduction, and effective strategies to prevent and treat metabolic liver disease can reduce incident HCC [230]. Implementation science is needed to improve surveillance adherence, ensure timely diagnostic workup, and reduce disparities in access to curative-intent therapy, such as Risk Stratification for Early-onset Colorectal Cancer Screening: Are We Ready for Implementation [231]. Real-world evidence can complement clinical trials by evaluating outcomes in broader populations and by informing sequencing and safety in patients with impaired hepatic reserve [232]. The evolving landscape of HCC treatment necessitates a continuous reevaluation of therapeutic strategies to optimize outcomes for diverse patient populations. The future of hepatocellular carcinoma management will increasingly rely on integrating innovative therapies with traditional approaches, particularly as new immunotherapeutic strategies emerge and evolve.

### 8.4. Integrative Care and Future Clinical Trials

Integrative strategies that combine evidence-based oncologic therapy with structured supportive care—including symptom-focused non-pharmacological interventions (e.g., acupuncture/acupressure) and, where culturally and clinically appropriate, standardized traditional medicine products—merit prospective evaluation. Future trials should prespecify hepatic safety monitoring, rigorous assessment of herb–drug interactions, and standardized product quality control. Pragmatic randomized designs in intermediate-stage or conversion-intent settings could test whether integrative supportive packages improve treatment tolerance, nutritional status, fatigue, and quality of life without compromising oncologic endpoints, while also enabling embedded health-economic and implementation analyses. Such studies would help define where integrative care adds measurable value within modern multidisciplinary HCC pathways.

## 9. Conclusions

HCC management has embarked on a journey characterized by a broader array of options and enhanced results. Curative-intent strategies remain pivotal, while advanced locoregional methods and contemporary systemic therapies—particularly those based on immunotherapy combinations—have led to improved survival rates and facilitated downstaging or conversion in certain patients. The primary challenge persists in harmonizing tumor control with the maintenance of hepatic function through agile, multidisciplinary care. Future advancements will rely on biomarker-driven customization, safer and more effective combinations, and fair implementation of prevention and early detection initiatives that reach the increasing at-risk population, especially in metabolic liver ailments. Incorporating assessments of frailty into clinical practice can improve decision-making and customize treatment strategies, ultimately leading to better outcomes for individuals with hepatocellular carcinoma. Merging innovative therapies with traditional methods is crucial for optimizing results in hepatocellular carcinoma, particularly as treatment strategies become more intricate and comprehensive. The integration of innovative therapies with traditional approaches will be essential in addressing the complexities of hepatocellular carcinoma, ultimately enhancing patient outcomes and survival rates.

## Figures and Tables

**Table 1:** Evaluation domains and clinical parameters for treatment decision-making in hepatocellular carcinoma.

Domain	Assessment Indicator	Clinical Category	Features and Notes
Liver function	Child–Pugh classification	A: suitable for most treatments B: limited eligibility C: BSC except transplant	The foundation for all treatment eligibility decisions Most clinical trials use only CP-A cases as controls Includes subjective items CP-B cases represent a heterogeneous group with significant prognosis variation
Liver function	ALBI grade	Grade 1–2: better tolerance Grade 3: high toxicity risk	Risk Stratification within CP-A Useful for assessing tolerability of TACE and systemic therapy Does not reflect presence of encephalopathy or ascites
Portal hypertension	Platelet count, varices, spleen size, ascites history; HVPG when available	HVPG ≥ 10 mmHg presence of varices	Directly linked to the risk of bleeding during surgery or anti-VEGF drug therapy HVPG measurement is difficult in general clinical practice
Frailty Sarcopenia	Grip strength, gait speed, CT skeletal muscle index, G8/VES-13 screening	Low muscle mass Functional decline	Setting various treatment intensities Determining the need for nutritional and exercise interventions
Renal/cardiovascular reserve	eGFR, proteinuria, BP, cardiac function	Decreased renal function Hypertension	Affects selection of systemic regimens, including anti-VEGF or TKI
Autoimmune history Transplant status	Autoimmune disease, immunosuppression, graft history	Suitability for immunotherapy	Critical factors for selecting between ICI and TKI

BSC, best supportive care; CP, Child–Pugh; ALBI, albumin-bilirubin; TACE, transcatheter arterial chemoembolization; HVPG, hepatic venous pressure gradient; VEGF, vascular endothelial growth factor; CT, computed tomography; G8, Geriatric-8; VES-13, Vulnerable Elders Survey-13; eGFR, estimated Glomerular Filtration Rate; BP, blood pressure; TKI, tyrosine kinase inhibitor.

**Table 2:** Overview of therapeutic modalities and practical considerations in hepatocellular carcinoma.

Treatment	Indication	Feature	Limitation
Surgical resection	BCLC 0/A Good liver function (CP-A, etc.) No portal hypertension	Can be expected to be cured	High recurrence rate Cases with poor hepatic reserve are not eligible
Ablation	Very early/early tumors ≤ 3 cm	Minimally invasive Repeatable Effective for transplant bridging	Depends on tumor location Requires caution near the bile duct
Liver transplantation	Within transplant criteria Selected downstaging	Curative strategies for high-risk cases Simultaneously address tumors and liver dysfunction	Organ availability Recurrence still possible
TACE	Intermediate-stage Liver-only disease	Proven survival benefits Widely available	Repeated TACE causes liver function impairment The existence of cases unsuitable for TACE
TARE	Intermediate–selected advanced Vascular invasion possible	Long-lasting effects Fewer treatment sessions required Can be performed even in PVTT cases	Specialized expertise Radiation liver injury risk
SBRT/Proton therapy	Local control when surgery/ablation not feasible	Precise and non-invasive Potential to reduce disease stage	Planning complexity Radiation liver injury risk
HAIC	Liver-dominant disease PVTT cases Rapid control needed when systemic limited	High intrahepatic drug concentration	Catheter-related complications
Systemic therapy	Advanced stage TACE-unsuitable BCLC B Mainly CP-A	Highest response rates Conversion possibilities	irAEs/bleeding risk The problem of healthcare costs

BCLC, Barcelona Clinic Liver Cancer; CP, Child–Pugh; TACE, transcatheter arterial chemoembolization; TARE, transarterial radioembolization; PVTT, portal vein tumor thrombosis; SBRT, stereotactic body radiotherapy; HAIC, hepatic arterial infusion chemotherapy; irAE, immune-related adverse events.

**Table 3:** Landmark randomized trials and key survival estimates across major hepatocellular carcinoma treatment modalities.

Key PFS/TTP Result	Key OS Result	Primary Endpoint	N (Randomized)	Arms (Comparison)	Trial (Year)	Setting/Line	Modality
mPFS 6.9 vs. 4.3 mo; HR 0.65	mOS 19.2 vs. 13.4 mo; HR 0.66	OS and PFS	501	Atezolizumab + Bevacizumab vs. Sorafenib	IMbrave150 (2022 upd.)	1 L advanced uHCC	Systemic
PFS not significantly different (per abstract).	mOS 16.43 vs. 13.77 mo; HR 0.78	OS	1171	STRIDE (Tremelimumab 1 dose + Durvalumab) vs. Sorafenib	HIMALAYA (2022)	1 L advanced uHCC	Systemic
mPFS 7.4 vs. 3.7 mo	mOS 13.6 vs. 12.3 mo; HR 0.92 (noninferior)	OS (noninferiority)	954	Lenvatinib vs. Sorafenib	REFLECT (2018)	1 L advanced uHCC	Systemic
mPFS 2.1 vs. 3.4 mo (favored sorafenib)	mOS 15.9 vs. 14.1 mo; HR 0.85 (noninferior)	OS (noninferiority)	674	Tislelizumab vs. Sorafenib	RATIONALE-301 (2023)	1 L advanced uHCC	Systemic
mPFS 4.6 vs. 2.8 mo; HR 0.56	mOS NR vs. 10.4 mo; HR 0.57 (interim)	OS and PFS	571	Sintilimab + IBI305 (bevacizumab biosimilar) vs. Sorafenib	ORIENT-32 (2021)	1 L advanced (China; HBV)	Systemic
mPFS 5.6 vs. 3.7 mo; HR 0.54	mOS 23.8 vs. 15.2 mo; HR 0.64	PFS and OS	543	Camrelizumab + Rivoceranib vs. Sorafenib	CARES-310 (2025)	1 L advanced uHCC	Systemic
mPFS 8.2 vs. 8.0 mo; HR 0.87 (NS per prespec.)	mOS 21.2 vs. 19.0 mo; HR 0.84 (NS per prespec.)	OS and PFS (superiority)	794	Lenvatinib + Pembrolizumab vs. Lenvatinib	LEAP-002 (2023)	1 L advanced uHCC	Systemic
mPFS 6.9 vs. 4.3 mo; HR 0.74	mOS 16.5 vs. 15.5 mo; HR 0.98 (NS)	OS (all) and PFS (subset; combo vs. sora)	649	Cabozantinib + Atezolizumab vs. Sorafenib	COSMIC-312 (final 2024)	1 L advanced uHCC	Systemic
Not primary; OS did not meet significance threshold.	mOS 16.4 vs. 14.7 mo; HR 0.85 (NS)	OS	743	Nivolumab vs. Sorafenib	CheckMate 459 (2022)	1 L advanced uHCC	Systemic
Time to radiologic progression improved (see paper).	mOS 10.7 vs. 7.9 mo; HR 0.69	OS	602	Sorafenib vs. Placebo	SHARP (2008)	1 L advanced uHCC	Systemic
TTP improved (see paper).	mOS 6.5 vs. 4.2 mo; HR 0.68	OS	226	Sorafenib vs. Placebo	Asia-Pacific (2009)	1 L advanced (Asia-Pacific)	Systemic
mPFS 3.1 vs. 1.5 mo	mOS 10.6 vs. 7.8 mo; HR 0.63	OS	573	Regorafenib vs. Placebo	RESORCE (2017)	2 L after sorafenib	Systemic
mPFS 5.2 vs. 1.9 mo	mOS 10.2 vs. 8.0 mo; HR 0.76	OS	707	Cabozantinib vs. Placebo	CELESTIAL (2018)	2 L/3 L after prior systemic	Systemic
See paper (PFS improved).	mOS 8.5 vs. 7.3 mo; HR 0.71	OS	292	Ramucirumab vs. Placebo	REACH-2 (2019)	2 L (AFP ≥ 400 ng/mL)	Systemic
Did not meet prespecified significance.	mOS 13.9 vs. 10.6 mo; HR 0.781 (did not meet prespec.)	OS and PFS	413	Pembrolizumab vs. Placebo (BSC)	KEYNOTE-240 (final 2023)	2 L (global)	Systemic
mPFS 2.6 vs. 2.3 mo	mOS 14.6 vs. 13.0 mo; HR 0.79	OS	453	Pembrolizumab vs. Placebo (BSC)	KEYNOTE-394 (2022/2023)	2 L (Asia)	Systemic
Response/QoL favored SIRT (see paper).	mOS 8.0 vs. 9.9 mo (NS)	OS	467	Y-90 SIRT vs. Sorafenib	SARAH (2017)	BCLC B/advanced (after TACE fail)	Locoregional
See paper.	mOS 8.8 vs. 10.0 mo; HR 1.1 (NS)	OS	360	Y-90 RE vs. Sorafenib	SIRveNIB (2018)	Advanced (Asia-Pacific)	Locoregional
Updated PFS 22.8 vs. 13.5 mo; HR 0.661		PFS (per protocol)	156	TACE + Sorafenib vs. TACE alone	TACTICS (2020; upd 2022)	Unresectable HCC (TACE-eligible)	Locoregional

Abbreviations: 1 L, first-line; uHCC, unresectable hepatocellular carcinoma; OS, overall survival; PFS, progression-free survival; TTP, time-to-progression; HR, hazard ratio; mOS, median overall survival; mPFS, median progression-free survival; NR, not reached.

## Data Availability

Not applicable.

## References

[1] Martinez-Chantar M.L., Avila M.A., Lu S.C. (2020). Hepatocellular Carcinoma: Updates in Pathogenesis, Detection and Treatment. Cancers.

[2] Bupathi M., Kaseb A., Meric-Bernstam F., Naing A. (2015). Hepatocellular carcinoma: Where there is unmet need. Mol. Oncol..

[3] D’Avola D., Granito A., Torre-Alaez M., Piscaglia F. (2022). The importance of liver functional reserve in the non-surgical treatment of hepatocellular carcinoma. J. Hepatol..

[4] Phipps M.M., Choi A.J., Brown R.S. (2024). Narrative review of management of cirrhosis during the treatment of hepatocellular carcinoma. Ann. Palliat. Med..

[5] Sakai N., Clarke C.N., Schuster R., Blanchard J., Tevar A.D., Edwards M.J., Lentsch A.B. (2010). Portal vein ligation accelerates tumor growth in ligated, but not contralateral lobes. World J. Gastroenterol..

[6] Mehta N. (2020). Hepatocellular Carcinoma-How to Determine Therapeutic Options. Hepatol. Commun..

[7] Cillo U., Gringeri E., D’Amico F.E., Lanari J., Furlanetto A., Vitale A. (2025). Hepatocellular carcinoma: Revising the surgical approach in light of the concept of multiparametric therapeutic hierarchy. Dig. Liver Dis..

[8] Daher D., Seif El Dahan K., Cano A., Gonzales M., Ransom C., Jaurez E., Carranza O., Quirk L., Morgan T., Gopal P. (2024). Hepatocellular Carcinoma Surveillance Patterns and Outcomes in Patients with Cirrhosis. Clin. Gastroenterol. Hepatol..

[9] Saito N., Tanaka T., Nishiohuku H., Sato T., Masada T., Matsumoto T., Anai H., Sakaguchi H., Sueyoshi S., Marugami N. (2020). Transarterial-chemoembolization remains an effective therapy for intermediate-stage hepatocellular carcinoma with preserved liver function. Hepatol. Res..

[10] Hiraoka A., Kumada T., Kudo M., Hirooka M., Koizumi Y., Hiasa Y., Tajiri K., Toyoda H., Tada T., Ochi H. (2017). Hepatic Function during Repeated TACE Procedures and Prognosis after Introducing Sorafenib in Patients with Unresectable Hepatocellular Carcinoma: Multicenter Analysis. Dig. Dis..

[11] European Association for the Study of the Liver (2025). EASL Clinical Practice Guidelines on the management of hepatocellular carcinoma. J. Hepatol..

[12] Vergoulidou M. (2015). More than a Decade of Tyrosine Kinase Inhibitors in the Treatment of Solid Tumors: What We Have Learned and What the Future Holds. Biomark. Insights.

[13] Zanuso V., Rimassa L., Braconi C. (2025). The rapidly evolving landscape of HCC: Selecting the optimal systemic therapy. Hepatology.

[14] Foy V., McNamara M.G., Valle J.W., Lamarca A., Edeline J., Hubner R.A. (2023). Current Evidence for Immune Checkpoint Inhibition in Advanced Hepatocellular Carcinoma. Curr. Oncol..

[15] Gish R.G. (2006). Hepatocellular carcinoma: Overcoming challenges in disease management. Clin. Gastroenterol. Hepatol..

[16] Carr B.I., Guerra V. (2013). HCC and its microenvironment. Hepatogastroenterology.

[17] Woo H.Y., Heo J. (2015). New perspectives on the management of hepatocellular carcinoma with portal vein thrombosis. Clin. Mol. Hepatol..

[18] Lee J.J.X., Tai D.W., Choo S.P. (2021). Locoregional therapy in hepatocellular carcinoma: When to start and when to stop and when to revisit. ESMO Open.

[19] Liu D., Imai N. (2025). Decision-Making Biomarkers Guiding Therapeutic Strategies in Hepatocellular Carcinoma: From Prediction to Personalized Care. Cancers.

[20] Heller M., Parikh N.D., Fidelman N., Owen D. (2021). Frontiers of therapy for hepatocellular carcinoma. Abdom. Radiol..

[21] Wallace M.C., Preen D., Jeffrey G.P., Adams L.A. (2015). The evolving epidemiology of hepatocellular carcinoma: A global perspective. Expert Rev. Gastroenterol. Hepatol..

[22] Jaber F., Cholankeril G., El-Serag H.B. (2024). Contemporary epidemiology of hepatocellular carcinoma: Understanding risk factors and surveillance strategies. J. Can. Assoc. Gastroenterol..

[23] Dhir M., Melin A.A., Douaiher J., Lin C., Zhen W.K., Hussain S.M., Geschwind J.F., Doyle M.B., Abou-Alfa G.K., Are C. (2016). A Review and Update of Treatment Options and Controversies in the Management of Hepatocellular Carcinoma. Ann. Surg..

[24] Hoo R., Chua K.L.M., Panda P.K., Skanderup A.J., Tan D.S.W. (2024). Precision Endpoints for Contemporary Precision Oncology Trials. Cancer Discov..

[25] Cortese F., Anagnostopoulos F., Bazzocchi M.V., Caringi S., Pisani A.R., Renzulli M., Paraskevopoulos I., Laera L., Surgo A., Spiliopoulos S. (2025). Modern approach to hepatocellular carcinoma treatment. World J. Hepatol..

[26] Veracruz N., Gish R.G., Cheung R., Chitnis A.S., Wong R.J. (2022). Global incidence and mortality of hepatitis B and hepatitis C acute infections, cirrhosis and hepatocellular carcinoma from 2010 to 2019. J. Viral. Hepat..

[27] Chen C.J. (2018). Global elimination of viral hepatitis and hepatocellular carcinoma: Opportunities and challenges. Gut.

[28] Wong G.L., Hui V.W., Yip T.C., Liang L.Y., Zhang X., Tse Y.K., Lai J.C., Chan H.L., Wong V.W. (2022). Universal HBV vaccination dramatically reduces the prevalence of HBV infection and incidence of hepatocellular carcinoma. Aliment. Pharmacol. Ther..

[29] Przybyszewski E.M., Chung R.T. (2023). Unmet Needs in the Post-Direct-Acting Antiviral Era: Hepatocarcinogenesis After Hepatitis C Virus Eradication. J. Infect. Dis..

[30] Ferdous S.E., Ferrell J.M. (2024). Pathophysiological Relationship between Type 2 Diabetes Mellitus and Metabolic Dysfunction-Associated Steatotic Liver Disease: Novel Therapeutic Approaches. Int. J. Mol. Sci..

[31] Omura T. (2025). “Older Adult Diabetes”: A Conceptual Proposal for a Distinct Clinical Entity. Diabetes Spectr..

[32] Shah P.A., Patil R., Harrison S.A. (2023). NAFLD-related hepatocellular carcinoma: The growing challenge. Hepatology.

[33] Tovo C.V., de Mattos A.Z., Coral G.P., Sartori G.D.P., Nogueira L.V., Both G.T., Villela-Nogueira C.A., de Mattos A.A. (2023). Hepatocellular carcinoma in non-alcoholic steatohepatitis without cirrhosis. World J. Gastroenterol..

[34] Merrill C., Samuel A., Gupta S., Wilson S.R. (2023). A Novel Technology for Resolution of CEUS Imaging Problems in Patients With High BMI and Fatty Liver. J. Ultrasound Med..

[35] Pashayan N., Pharoah P.D.P. (2020). The challenge of early detection in cancer. Science.

[36] Levrero M., Zucman-Rossi J. (2016). Mechanisms of HBV-induced hepatocellular carcinoma. J. Hepatol..

[37] Mason W.S., Jilbert A.R., Litwin S. (2021). Hepatitis B Virus DNA Integration and Clonal Expansion of Hepatocytes in the Chronically Infected Liver. Viruses.

[38] Lin D., Yang H.I., Nguyen N., Hoang J., Kim Y., Vu V., Le A., Chaung K., Nguyen V., Trinh H. (2016). Reduction of chronic hepatitis B-related hepatocellular carcinoma with anti-viral therapy, including low risk patients. Aliment. Pharmacol. Ther..

[39] Zampino R., Pisaturo M.A., Cirillo G., Marrone A., Macera M., Rinaldi L., Stanzione M., Durante-Mangoni E., Gentile I., Sagnelli E. (2015). Hepatocellular carcinoma in chronic HBV-HCV co-infection is correlated to fibrosis and disease duration. Ann. Hepatol..

[40] Semmler G., Binter T., Kozbial K., Schwabl P., Hametner-Schreil S., Zanetto A., Gavasso S., Chromy D., Bauer D.J.M., Simbrunner B. (2021). Noninvasive Risk Stratification After HCV Eradication in Patients with Advanced Chronic Liver Disease. Hepatology.

[41] Ito T., Nguyen M.H. (2023). Perspectives on the Underlying Etiology of HCC and Its Effects on Treatment Outcomes. J. Hepatocell. Carcinoma.

[42] Jacob R., Prince D.S., Kench C., Liu K. (2023). Alcohol and its associated liver carcinogenesis. J. Gastroenterol. Hepatol..

[43] Hofer B.S., Simbrunner B., Hartl L., Jachs M., Bauer D.J.M., Balcar L., Paternostro R., Schwabl P., Semmler G., Scheiner B. (2023). Alcohol Abstinence Improves Prognosis Across All Stages of Portal Hypertension in Alcohol-Related Cirrhosis. Clin. Gastroenterol. Hepatol..

[44] Mahle R., McLean Diaz P., Marshall C., Goodman R.P., Schaefer E., Luther J. (2023). Integrated hepatology and addiction care for inpatients with alcohol use disorder improves outcomes: A prospective study. Hepatol. Commun..

[45] Habib S. (2024). Metabolic dysfunction-associated steatotic liver disease heterogeneity: Need of subtyping. World J. Gastrointest. Pathophysiol..

[46] Gupta T. (2022). Nonalcoholic steatohepatitis and hepatocellular carcinoma: Beyond the boundaries of the liver. World J. Gastroenterol..

[47] Bader H., Yamin S., Alshahwan H., Farraj H., Maghnam J., Abu Omar Y. (2024). Association between Metabolic-Dysfunction-Associated Steatotic Liver Disease and Hepatic Cancer: Current Concepts and Future Challenges. J. Clin. Med..

[48] Koo E., Singal A.G. (2024). Hepatocellular Carcinoma Surveillance: Evidence-Based Tailored Approach. Surg. Oncol. Clin. N. Am..

[49] Faria S.C., Sagebiel T., Patnana M., Cox V., Viswanathan C., Lall C., Qayyum A., Bhosale P.R. (2019). Tumor markers: Myths and facts unfolded. Abdom. Radiol..

[50] Esfeh J.M., Hajifathalian K., Ansari-Gilani K. (2020). Sensitivity of ultrasound in detecting hepatocellular carcinoma in obese patients compared to explant pathology as the gold standard. Clin. Mol. Hepatol..

[51] Stamilio D., Carlson L.M. (2016). Transabdominal ultrasound is appropriate. Am. J. Obstet. Gynecol..

[52] Le Q.A., Kiener T., Johnson H.A., Li K.H., Limburg P.J., Fendrick A.M., Kisiel J.B., Ebner D.W. (2025). Adherence to recommended blood-based screening tests for cancer and chronic diseases: A systematic literature review. Prev. Med..

[53] Noureddin N., Copur-Dahi N., Loomba R. (2024). Monitoring disease progression in metabolic dysfunction-associated steatotic liver disease. Aliment. Pharmacol. Ther..

[54] Aberg F., Saarinen K., Jula A., Lundqvist A., Vihervaara T., Erlund I., Farkkila M. (2023). Combined use of the ELF test and CLivD score improves prediction of liver-related outcomes in the general population. Liver Int..

[55] Brugada P. (2021). On risk stratification and its paradoxes. Eur. Heart J..

[56] Sherman M. (2019). How to improve HCC surveillance outcomes. JHEP Rep..

[57] Chavez-Villa M., Dominguez-Rosado I. (2024). Overview of Current Hepatocellular Carcinoma Staging Systems: Is There an Optimal System?. Surg. Oncol. Clin. N. Am..

[58] Kohla M., Ashour R., Taha H., El-Abd O., Osman M., Abozeid M., ELKhadry S.W. (2024). Prognostic performance of Hong Kong Liver Cancer with Barcelona Clinic Liver Cancer staging systems in hepatocellular carcinoma. BMC Gastroenterol..

[59] Zheng R., Busemeyer J.R., Nosofsky R.M. (2023). Integrating Categorization and Decision-Making. Cogn. Sci..

[60] Vitale A., Cabibbo G., Iavarone M., Vigano L., Pinato D.J., Ponziani F.R., Lai Q., Casadei-Gardini A., Celsa C., Galati G. (2023). Personalised management of patients with hepatocellular carcinoma: A multiparametric therapeutic hierarchy concept. Lancet Oncol..

[61] Sharma P. (2022). Value of Liver Function Tests in Cirrhosis. J. Clin. Exp. Hepatol..

[62] Sanders S., Flaws D., Than M., Pickering J.W., Doust J., Glasziou P. (2016). Simplification of a scoring system maintained overall accuracy but decreased the proportion classified as low risk. J. Clin. Epidemiol..

[63] Xu S.X., Yang F., Ge N., Guo J.T., Sun S.Y. (2024). Role of albumin-bilirubin score in non-malignant liver disease. World J. Gastroenterol..

[64] Piscaglia F., La Mura V., Ravaioli F. (2023). A pragmatic strategy for the screening and treatment of portal hypertension in patients needing systemic treatment for advanced hepatocellular carcinoma. Dig. Liver Dis..

[65] de Franchis R., Dell’Era A. (2014). Invasive and noninvasive methods to diagnose portal hypertension and esophageal varices. Clin. Liver Dis..

[66] Hernandez-Gea V., Berzigotti A. (2015). Clinical Evaluation and Prognosis. Dig. Dis..

[67] Cabibbo G., Aghemo A., Lai Q., Masarone M., Montagnese S., Ponziani F.R., Italian Association for the Study of the Liver (AISF) (2022). Optimizing systemic therapy for advanced hepatocellular carcinoma: The key role of liver function. Dig. Liver Dis..

[68] Fekete Z., Fekete A., Kacso G. (2024). Treatment Classification by Intent in Oncology-The Need for Meaningful Definitions: Curative, Palliative and Potentially Life-Prolonging. J. Pers. Med..

[69] Yacoub J.H., Hsu C.C., Fishbein T.M., Mauro D., Moon A., He A.R., Bashir M.R., Burke L.M.B. (2021). Therapies for hepatocellular carcinoma: Overview, clinical indications, and comparative outcome evaluation-part one: Curative intention. Abdom. Radiol..

[70] Romagnoli R., Mazzaferro V., Bruix J. (2015). Surgical resection for hepatocellular carcinoma: Moving from what can be done to what is worth doing. Hepatology.

[71] Llovet J.M., Schwartz M., Fuster J., Bruix J. (2006). Expanded criteria for hepatocellular carcinoma through down-staging prior to liver transplantation: Not yet there. Semin. Liver Dis..

[72] Llovet J.M., De Baere T., Kulik L., Haber P.K., Greten T.F., Meyer T., Lencioni R. (2021). Locoregional therapies in the era of molecular and immune treatments for hepatocellular carcinoma. Nat. Rev. Gastroenterol. Hepatol..

[73] Yasui Y., Tsuchiya K., Kurosaki M., Takeguchi T., Takeguchi Y., Okada M., Wang W., Kubota Y., Goto T., Komiyama Y. (2018). Up-to-seven criteria as a useful predictor for tumor downstaging to within Milan criteria and Child-Pugh grade deterioration after initial conventional transarterial chemoembolization. Hepatol. Res..

[74] Tsujita Y., Sofue K., Ueshima E., Ueno Y., Hori M., Tsurusaki M., Murakami T. (2023). Evaluation and Prediction of Treatment Response for Hepatocellular Carcinoma. Magn. Reson. Med. Sci..

[75] Liao J.I., Ho S.Y., Hou M.C., Liu P.H., Hsu C.Y., Huo T.I. (2024). Performance status as a prognostic surrogate in hepatocellular carcinoma: Role of albumin-bilirubin and easy-albumin-bilirubin grade. J. Chin. Med. Assoc..

[76] Oura K., Morishita A., Manabe T., Takuma K., Nakahara M., Tadokoro T., Fujita K., Mimura S., Tani J., Ono M. (2023). Relationship between Accurate Diagnosis of Sarcopenia and Prognosis in Patients with Hepatocellular Carcinoma Treated with Atezolizumab plus Bevacizumab Combination Therapy. Cancers.

[77] Faruque L.I., Lin M., Battistella M., Wiebe N., Reiman T., Hemmelgarn B., Thomas C., Tonelli M. (2014). Systematic review of the risk of adverse outcomes associated with vascular endothelial growth factor inhibitors for the treatment of cancer. PLoS ONE.

[78] Cammarota A., D’Alessio A., Pressiani T., Rimassa L., Personeni N. (2021). Systemic Treatment for Older Patients with Unresectable Hepatocellular Carcinoma. Drugs Aging.

[79] Brunot A., Le Sourd S., Pracht M., Edeline J. (2016). Hepatocellular carcinoma in elderly patients: Challenges and solutions. J. Hepatocell. Carcinoma.

[80] Deng L., Yang C., Li L.Q., Zhong J.H. (2015). Hepatic Resection Improves Long-Term Survival of Patients with Large and/or Multinodular Hepatocellular Carcinoma. J. Gastrointest. Surg..

[81] Aydin C., Yilmaz S. (2020). Is Macroscopic Portal Vein Tumor Thrombosis of HCC Really an Exclusion for Liver Transplantation?. J. Gastrointest. Cancer.

[82] Tampaki M., Papatheodoridis G.V., Cholongitas E. (2021). Intrahepatic recurrence of hepatocellular carcinoma after resection: An update. Clin. J. Gastroenterol..

[83] Polvieng T., Hongjinda S., Thienhiran A., Burasakarn P., Fuengfoo P. (2024). Effect of Sarcopenia on the Prognosis of Clinical Outcomes in Patients with Hepatocellular Carcinoma After Hepatic Resection. Am. Surg..

[84] Fu Y., Li X., Yang Z., Li S., Pan Y., Chen J., Wang J., Hu D., Zhou Z., Xu L. (2023). A risk-based postresection follow-up strategy for hepatocellular carcinoma patients. Cancer.

[85] von Minckwitz G., Loibl S., Maisch A., Untch M. (2011). Lessons from the neoadjuvant setting on how best to choose adjuvant therapies. Breast.

[86] Maruyama H., Tobari M., Nagamatsu H., Yamaguchi T., Shiina S. (2023). Ablation for Benign Liver Tumors: Current Concepts and Limitations. J. Clin. Transl. Hepatol..

[87] Ergun O., Elshamy M., Berber E. (2022). Ablation technologies. Surg. Open Sci..

[88] Imajo K., Ogawa Y., Yoneda M., Saito S., Nakajima A. (2020). A review of conventional and newer generation microwave ablation systems for hepatocellular carcinoma. J. Med. Ultrason..

[89] Jarnagin W.R., D’Angelica M.I. (2022). Advances in the management of liver and biliary tumors. J. Surg. Oncol..

[90] Ponniah S.A., Zori A.G., Cabrera R., Sergi C.M. (2021). Locoregional Therapies for Bridging and Downstaging Hepatocellular Carcinoma Prior to Liver Transplant. Liver Cancer.

[91] Papaconstantinou D., Tsilimigras D.I., Pawlik T.M. (2022). Recurrent Hepatocellular Carcinoma: Patterns, Detection, Staging and Treatment. J. Hepatocell. Carcinoma.

[92] Campbell W.A., Makary M.S. (2024). Advances in Image-Guided Ablation Therapies for Solid Tumors. Cancers.

[93] Hill A., Olumba F., Chapman W. (2024). Transplantation for Hepatocellular Carcinoma. Surg. Clin. N. Am..

[94] Altundag O. (2022). Solid-Organ Transplantation from Deceased and Living Donors with Cancer or a History of Cancer. Exp. Clin. Transplant..

[95] Yin C., Armstrong S., Shin R., Geng X., Wang H., Satoskar R.S., Fishbein T., Smith C., Banovac F., Kim A.Y. (2023). Bridging and downstaging with TACE in early and intermediate stage hepatocellular carcinoma: Predictors of receiving a liver transplant. Ann. Gastroenterol. Surg..

[96] Lai Q., Lesari S., Lerut J.P. (2022). The impact of biological features for a better prediction of posttransplant hepatocellular cancer recurrence. Curr. Opin. Organ. Transplant..

[97] Giuliani T., Montalva E., Maupoey J., Bosca A., Hernando A., Calatayud D., Navarro V., Rubin A., Vinaixa C., Lopez-Andujar R. (2024). Recurrence of Hepatocellular Carcinoma after Liver Transplantation: Clinical Patterns and Hierarchy of Salvage Treatments. Dig. Surg..

[98] Jones-Pauley M., Victor D.W., Kodali S. (2024). Pushing the limits of treatment for hepatocellular carcinoma. Curr. Opin. Organ. Transplant..

[99] Lanza C., Ascenti V., Amato G.V., Pellegrino G., Triggiani S., Tintori J., Intrieri C., Angileri S.A., Biondetti P., Carriero S. (2025). All You Need to Know About TACE: A Comprehensive Review of Indications, Techniques, Efficacy, Limits, and Technical Advancement. J. Clin. Med..

[100] Ji K., Shi Y., Liang Z., Zhang C., Jing L., Xu T., Cao S., Zhou G., Cao Y., Niu J. (2024). Lipiodol Combined with Drug-eluting Beads Versus Drug-eluting Beads Alone for Transarterial Chemoembolization of Hepatocellular carcinoma: A Multicenter Study. Acad. Radiol..

[101] Ben Ammar M., Nouri-Neuville M., Cornelis F.H. (2019). Percutaneous image-guided therapies of primary liver tumors: Techniques and outcomes. Presse. Med..

[102] Burley C.G., Kumar M.H., Bhatti W.A., Boyd C., Sales C.M. (2019). Transcaval embolization as the preferred approach. J. Vasc. Surg..

[103] Sueyoshi E., Hayashida T., Sakamoto I., Uetani M. (2010). Vascular complications of hepatic artery after transcatheter arterial chemoembolization in patients with hepatocellular carcinoma. AJR Am. J. Roentgenol..

[104] Ghabili K., Windham-Herman A.M., Konstantinidis M., Murali N., Borde T., Adam L.C., Laage-Gaupp F., Lin M., Chapiro J., Georgiades C. (2024). Outcomes of repeat conventional transarterial chemoembolization in patients with liver metastases. Ann. Hepatol..

[105] Muller L., Stoehr F., Mahringer-Kunz A., Hahn F., Weinmann A., Kloeckner R. (2021). Current Strategies to Identify Patients That Will Benefit from TACE Treatment and Future Directions a Practical Step-by-Step Guide. J. Hepatocell. Carcinoma.

[106] Pesapane F., Nezami N., Patella F., Geschwind J.F. (2017). New concepts in embolotherapy of HCC. Med. Oncol..

[107] Sacco R., Conte C., Tumino E., Parisi G., Marceglia S., Metrangolo S., Eggenhoffner R., Bresci G., Cabibbo G., Giacomelli L. (2016). Transarterial radioembolization for hepatocellular carcinoma: A review. J. Hepatocell. Carcinoma.

[108] Badar W., Yu Q., Patel M., Ahmed O. (2024). Transarterial Radioembolization for Management of Hepatocellular Carcinoma. Oncologist.

[109] Yu Q., Khanjyan M., Fidelman N., Pillai A. (2023). Contemporary applications of Y90 for the treatment of hepatocellular carcinoma. Hepatol. Commun..

[110] Boas F.E., Maxwell A.W.P. (2023). Beyond Mean Tumor Dose: The Importance of Particle Density in Radioembolization. J. Vasc. Interv. Radiol..

[111] Salem R., Padia S.A., Toskich B.B., Callahan J.D., Fowers K.D., Geller B.S., Johnson G.E., Kulik L., Patel T.C., Lewandowski R.J. (2025). Radiation segmentectomy for early hepatocellular carcinoma is curative. J. Hepatol..

[112] Lewis S., Dawson L., Barry A., Stanescu T., Mohamad I., Hosni A. (2022). Stereotactic body radiation therapy for hepatocellular carcinoma: From infancy to ongoing maturity. JHEP Rep..

[113] Roberts H.J., Wo J.Y. (2022). Stereotactic body radiation therapy for primary liver tumors: An effective liver-directed therapy in the toolbox. Cancer.

[114] Molitoris J.K. (2023). Start Combining, Stop Comparing. Int. J. Radiat. Oncol. Biol. Phys..

[115] Li J.X., Zhang R.J., Qiu M.Q., Yan L.Y., He M.L., Long M.Y., Zhong J.H., Lu H.Y., Zhou H.M., Xiang B.D. (2023). Non-classic radiation-induced liver disease after intensity-modulated radiotherapy for Child-Pugh grade B patients with locally advanced hepatocellular carcinoma. Radiat. Oncol..

[116] Miljanic M., Montalvo S., Aliru M., Song T., Leon-Camarena M., Innella K., Vujovic D., Komaki R., Iyengar P. (2022). The Evolving Interplay of SBRT and the Immune System, along with Future Directions in the Field. Cancers.

[117] Xu W., Li Q., Liang B. (2025). Hepatic Artery Infusion Chemotherapy for Hepatocellular Carcinoma: Clinical Advancements. Curr. Oncol..

[118] Dillon P.A., Foglia R.P. (2006). Complications associated with an implantable vascular access device. J. Pediatr. Surg..

[119] Tulpule V., Ballas L.K. (2021). Concomitant Systemic Therapy: Current and Future Perspectives. Clin. Oncol. R. Coll. Radiol..

[120] Dias E.S.D., Borad M., Uson Junior P.L.S. (2024). Current efficacy of hepatic arterial infusion chemotherapy in hepatocellular carcinoma. World J. Gastrointest. Oncol..

[121] He N., Jiang J. (2024). Contribution of immune cells in synergistic anti-tumor effect of ablation and immunotherapy. Transl. Oncol..

[122] Bent E.H., Wehrenberg-Klee E., Koay E.J., Goyal L., Wo J.Y. (2021). Integration of Systemic and Liver-Directed Therapies for Locally Advanced Hepatocellular Cancer: Harnessing Potential Synergy for New Therapeutic Horizons. J. Natl. Compr. Cancer Netw..

[123] Narayanan G., Koethe Y., Gentile N. (2024). Irreversible Electroporation of the Hepatobiliary System: Current Utilization and Future Avenues. Medicina.

[124] Belfiore M.P., De Chiara M., Reginelli A., Clemente A., Urraro F., Grassi R., Belfiore G., Cappabianca S. (2022). An overview of the irreversible electroporation for the treatment of liver metastases: When to use it. Front. Oncol..

[125] Nakai Y. (2024). Endoscopic Ultrasound-Guided Antitumor Therapy. Gastrointest. Endosc. Clin. N. Am..

[126] Pillai A.A., Ramanathan M., Kulik L. (2020). Locoregional Therapies for Hepatocellular Carcinoma: What Has Changed in the Past Ten Years?. Clin. Liver Dis..

[127] Nebelung H., Wolf T., Bund S., Radosa C.G., Plodeck V., Grosche-Schlee S., Riediger C., Hoffmann R.T., Kuhn J.P. (2021). Radioembolization versus portal vein embolization for contralateral liver lobe hypertrophy: Effect of cirrhosis. Abdom. Radiol..

[128] Entezari P., Gabr A., Kennedy K., Salem R., Lewandowski R.J. (2021). Radiation Lobectomy: An Overview of Concept and Applications, Technical Considerations, Outcomes. Semin. Interv. Radiol..

[129] Silk T., Silk M., Wu J. (2022). Up to seven criteria in selection of systemic therapy for hepatocellular carcinoma. World J. Gastroenterol..

[130] Zhang Y., Brekken R.A. (2022). Direct and indirect regulation of the tumor immune microenvironment by VEGF. J. Leukoc. Biol..

[131] Altundag O. (2024). Recent Advances in Systemic Therapy for Hepatocellular Carcinoma. Exp. Clin. Transplant..

[132] Wong S.K., Beckermann K.E., Johnson D.B., Das S. (2021). Combining anti-cytotoxic T-lymphocyte antigen 4 (CTLA-4) and -programmed cell death protein 1 (PD-1) agents for cancer immunotherapy. Expert Opin. Biol. Ther..

[133] Balsano R., Pino M., Bocchero E., Valenzi E., Pressiani T., Bozzarelli S., Rimassa L. (2025). Combining VEGF and PD-1/PD-L1 inhibition in advanced hepatocellular carcinoma: Clinical trials, real-world evidence, and future directions. Expert Opin. Biol. Ther..

[134] Chen Y., Han H., Cheng J., Cheng Q., Zhu S., Zhan P., Liu H., Song Y., Lv T. (2024). Efficacy and safety of anti-PD-1/PD-L1-based dual immunotherapies versus PD-1/PD-L1 inhibitor alone in patients with advanced solid tumor: A systematic review and meta-analysis. Cancer Immunol. Immunother..

[135] Starzer A.M., Wolff L., Popov P., Kiesewetter B., Preusser M., Berghoff A.S. (2024). The more the merrier? Evidence and efficacy of immune checkpoint- and tyrosine kinase inhibitor combinations in advanced solid cancers. Cancer Treat. Rev..

[136] Qin S., Li A., Yi M., Yu S., Zhang M., Wu K. (2019). Recent advances on anti-angiogenesis receptor tyrosine kinase inhibitors in cancer therapy. J. Hematol. Oncol..

[137] Kibrit J., Khan R., Jung B.H., Koppe S. (2018). Clinical Assessment and Management of Portal Hypertension. Semin. Intervent. Radiol..

[138] Pathak S., Sonbol M.B. (2021). Second-Line Treatment Options for Hepatocellular Carcinoma: Current Landscape and Future Direction. J. Hepatocell. Carcinoma.

[139] Peeters F., Dekervel J. (2023). Considerations for individualized first-line systemic treatment in advanced hepatocellular carcinoma. Curr. Opin. Pharmacol..

[140] Furukawa Y., Long D.E., Ellsworth S.G. (2020). Functional liver-image guided hepatic therapy (FLIGHT): A technique to maximize hepatic functional reserve. Med. Dosim..

[141] Ly A., Cheng H.H., Alwan L. (2019). Hepatitis C infection and chemotherapy toxicity. J. Oncol. Pharm. Pract..

[142] Carrion A.F., Martin P. (2021). Keeping Patients with End-Stage Liver Disease Alive While Awaiting Transplant: Management of Complications of Portal Hypertension. Clin. Liver Dis..

[143] Sherry A.D., Lin T.A., McCaw Z.R., Beck E.J., Kouzy R., Abi Jaoude J., Passy A.H., Miller A.M., Kupferman G.S., Fuller C.D. (2024). Improving the clinical meaning of surrogate endpoints: An empirical assessment of clinical progression in phase III oncology trials. Int. J. Cancer.

[144] Iwasa S., Kudo T., Takahari D., Hara H., Kato K., Satoh T. (2020). Practical guidance for the evaluation of disease progression and the decision to change treatment in patients with advanced gastric cancer receiving chemotherapy. Int. J. Clin. Oncol..

[145] Zhao Y., Wu D., Yao Q., Yuan H., Hu H., Li H. (2024). Progression patterns in patients with advanced hepatocellular carcinoma treated with local therapy, targeted drugs, and PD-1/PD-L1 inhibitors. Cent. Eur. J. Immunol..

[146] Chai N.X., Chapiro J. (2020). Therapy of Intermediate-Stage Hepatocellular Carcinoma: Current Evidence and Clinical Practice. Semin. Intervent. Radiol..

[147] Wang W., Zhao Y., Bai W., Han G. (2015). Response assessment for HCC patients treated with repeated TACE: The optimal time-point is still an open issue. J. Hepatol..

[148] Xu Q., Leng B., You R., Diao L., Wang C., Yu Z., Yin G. (2025). Transarterial chemoembolization for advanced hepatocellular carcinoma after failure of first-line systemic treatment: A single-center case series. Discov. Oncol..

[149] Zhang S., Wang W.S., Zhong B.Y., Ni C.F. (2022). Subsequent Treatment after Transarterial Chemoembolization Failure/Refractoriness: A Review Based on Published Evidence. J. Clin. Transl. Hepatol..

[150] Cerreto M., Cardone F., Cerrito L., Stella L., Santopaolo F., Pallozzi M., Gasbarrini A., Ponziani F.R. (2023). The New Era of Systemic Treatment for Hepatocellular Carcinoma: From the First Line to the Optimal Sequence. Curr. Oncol..

[151] Wang M.D., Xu X.J., Wang K.C., Diao Y.K., Xu J.H., Gu L.H., Yao L.Q., Li C., Lv G.Y., Yang T. (2024). Conversion therapy for advanced hepatocellular carcinoma in the era of precision medicine: Current status, challenges and opportunities. Cancer Sci..

[152] Karachaliou G.S., Dimitrokallis N., Moris D.P. (2024). Downstaging strategies for unresectable hepatocellular carcinoma. World J. Gastroenterol..

[153] Wang J., Hu Y., Zhou L., Yang Y., Chen J., Chen H., Wang H. (2024). Non-surgery strategy versus hepatectomy in hepatocellular carcinoma patients with complete response after conversion therapy: A meta-analysis. World J. Surg. Oncol..

[154] She W.H., Cheung T.T. (2024). Options and survival benefits of conversion therapy for unresectable hepatocellular carcinoma. World J. Gastroenterol..

[155] Haber P.K., Krenzien F., Saribeyoglu K., Pratschke J., Schoning W. (2024). Integrating the new systemic treatment landscape and surgical therapy in hepatocellular carcinoma. Turk. J. Surg..

[156] Cammarota A., Zanuso V., Pressiani T., Personeni N., Rimassa L. (2022). Assessment and Monitoring of Response to Systemic Treatment in Advanced Hepatocellular Carcinoma: Current Insights. J. Hepatocell. Carcinoma.

[157] Chen Y., Yang C., Sheng L., Jiang H., Song B. (2023). The Era of Immunotherapy in Hepatocellular Carcinoma: The New Mission and Challenges of Magnetic Resonance Imaging. Cancers.

[158] Han X., Sun Q., Xu M., Zhu G., Gao R., Ni B., Li J. (2023). Unraveling the Complexities of Immune Checkpoint Inhibitors in Hepatocellular Carcinoma. Semin. Liver Dis..

[159] Colloca G.A., Venturino A. (2024). Radiographic and serologic response in patients with unresectable hepatocellular carcinoma receiving systemic antineoplastic treatments: A trial-level analysis. Cancer.

[160] Kaneko S., Tsuchiya K., Yasui Y., Inada K., Kirino S., Yamashita K., Osawa L., Hayakawa Y., Sekiguchi S., Higuchi M. (2020). Strategy for advanced hepatocellular carcinoma based on liver function and portal vein tumor thrombosis. Hepatol. Res..

[161] Wang J.C., Xia A.L., Xu Y., Lu X.J. (2019). Comprehensive treatments for hepatocellular carcinoma with portal vein tumor thrombosis. J. Cell. Physiol..

[162] Roberts L.N. (2023). How to manage hemostasis in patients with liver disease during interventions. Hematol. Am. Soc. Hematol. Educ. Program.

[163] Verso M., Munoz A., Agnelli G. (2023). Bleeding risk with concomitant administration of VEGF-TKIs and anticoagulant agents. Semin. Oncol..

[164] Tellez L., Guerrero A., Albillos Martinez A. (2023). Treatment of portal vein thrombosis in cirrhosis patients. Rev. Esp. Enferm. Dig..

[165] Hanif H., Ali M.J., Susheela A.T., Khan I.W., Luna-Cuadros M.A., Khan M.M., Lau D.T. (2022). Update on the applications and limitations of alpha-fetoprotein for hepatocellular carcinoma. World J. Gastroenterol..

[166] Qadeer M.A., Abbas Z., Amjad S., Shahid B., Altaf A., Siyal M. (2024). Des-gamma-carboxy prothrombin and alpha-fetoprotein levels as biomarkers for hepatocellular carcinoma and their correlation with radiological characteristics. World J. Gastrointest. Pathophysiol..

[167] Al-Hasan M., Mehta N., Yang J.D., Singal A.G. (2025). Role of biomarkers in the diagnosis and management of HCC. Liver Transpl..

[168] Yang Z., Fu Y., Wang Q., Pan Y., Wang J., Chen J., Hu D., Zhou Z., Chen M., Zhang Y. (2025). Dynamic changes of serum alpha-fetoprotein predict the prognosis of bevacizumab plus immunotherapy in hepatocellular carcinoma. Int. J. Surg..

[169] Kielar A., Fowler K.J., Lewis S., Yaghmai V., Miller F.H., Yarmohammadi H., Kim C., Chernyak V., Yokoo T., Meyer J. (2018). Locoregional therapies for hepatocellular carcinoma and the new LI-RADS treatment response algorithm. Abdom. Radiol..

[170] Yuan Z., Zhang J., Yang H., Ye X.D., Xu L.C., Li W.T. (2016). Diffusion-Weighted MR Imaging of Hepatocellular Carcinoma: Current Value in Clinical Evaluation of Tumor Response to Locoregional Treatment. J. Vasc. Interv. Radiol..

[171] Ye X.D., Yuan Z., Zhang J., Yuan Z. (2017). Radiological biomarkers for assessing response to locoregional therapies in hepatocellular carcinoma: From morphological to functional imaging (Review). Oncol. Rep..

[172] Sheng R., Jin K., Sun W., Gao S., Zhang Y., Wu D., Zeng M. (2023). Prediction of therapeutic response of advanced hepatocellular carcinoma to combined targeted immunotherapy by MRI. Magn. Reson. Imaging.

[173] Zheng W., Chen X., Xiong M., Zhang Y., Song Y., Cao D. (2024). Clinical-Radiologic Morphology-Radiomics Model on Gadobenate Dimeglumine-Enhanced MRI for Identification of Highly Aggressive Hepatocellular Carcinoma: Temporal Validation and Multiscanner Validation. J. Magn. Reson. Imaging.

[174] Nakamura M., Chiba T., Kanayama K., Kanzaki H., Saito T., Kusakabe Y., Kato N. (2019). Epigenetic dysregulation in hepatocellular carcinoma: An up-to-date review. Hepatol. Res..

[175] Shibata T. (2021). Genomic landscape of hepatocarcinogenesis. J. Hum. Genet..

[176] Demory A., Nault J.C. (2020). Molecular perspectives for the treatment of hepatocellular carcinoma. Acta Gastroenterol. Belg..

[177] Campani C., Zucman-Rossi J., Nault J.C. (2023). Genetics of Hepatocellular Carcinoma: From Tumor to Circulating DNA. Cancers.

[178] Kim E. (2023). Tumor Immune Microenvironment as a New Therapeutic Target for Hepatocellular Carcinoma Development. Dev. Reprod..

[179] Bao R., Stapor D., Luke J.J. (2020). Molecular correlates and therapeutic targets in T cell-inflamed versus non-T cell-inflamed tumors across cancer types. Genome. Med..

[180] Cho S.F., Anderson K.C., Tai Y.T. (2022). Microenvironment Is a Key Determinant of Immune Checkpoint Inhibitor Response. Clin. Cancer Res..

[181] Di Ceglie I., Carnevale S., Rigatelli A., Grieco G., Molisso P., Jaillon S. (2024). Immune cell networking in solid tumors: Focus on macrophages and neutrophils. Front. Immunol..

[182] Sankar K., Ye J.C., Li Z., Zheng L., Song W., Hu-Lieskovan S. (2022). The role of biomarkers in personalized immunotherapy. Biomark. Res..

[183] Zhou X., Xing Z., Dong R., Zhang X., Liang X., Lu Z., Yang G. (2025). Cell Function Experiments and Bioinformatics Analysis Jointly Revealed the Antineoplastic Effect of Lumican on Hepatocellular Carcinoma. Phenomics.

[184] Chan Y.T., Zhang C., Wu J., Lu P., Xu L., Yuan H., Feng Y., Chen Z.S., Wang N. (2024). Biomarkers for diagnosis and therapeutic options in hepatocellular carcinoma. Mol. Cancer.

[185] Krebs M.G., Malapelle U., Andre F., Paz-Ares L., Schuler M., Thomas D.M., Vainer G., Yoshino T., Rolfo C. (2022). Practical Considerations for the Use of Circulating Tumor DNA in the Treatment of Patients with Cancer: A Narrative Review. JAMA Oncol..

[186] Gruson D., Bodovitz S. (2010). Rapid emergence of multimarker strategies in laboratory medicine. Biomarkers.

[187] Aquino I.M.C., Pascut D. (2024). Liquid biopsy: New opportunities for precision medicine in hepatocellular carcinoma care. Ann. Hepatol..

[188] Yoon S., Kim Y.J., Jeon J.S., Ahn S.J., Choi S.J. (2024). Radiomics and machine learning analysis of liver magnetic resonance imaging for prediction and early detection of tumor response in colorectal liver metastases. Korean J. Clin. Oncol..

[189] Mungenast F., Fernando A., Nica R., Boghiu B., Lungu B., Batra J., Ecker R.C. (2021). Next-Generation Digital Histopathology of the Tumor Microenvironment. Genes.

[190] Yu B., Ma W. (2024). Biomarker discovery in hepatocellular carcinoma (HCC) for personalized treatment and enhanced prognosis. Cytokine Growth Factor Rev..

[191] Samala R.K., Drukker K., Shukla-Dave A., Chan H.P., Sahiner B., Petrick N., Greenspan H., Mahmood U., Summers R.M., Tourassi G. (2024). AI and machine learning in medical imaging: Key points from development to translation. BJR Artif. Intell..

[192] Feng N., Wang K., Jiao Y. (2025). Integrating radiomics and machine learning for the diagnosis and prognosis of hepatocellular carcinoma. World J. Gastrointest. Oncol..

[193] Guan H., Zhang X., Kuang M., Yu J. (2022). The gut-liver axis in immune remodeling of hepatic cirrhosis. Front. Immunol..

[194] Bajaj J.S. (2019). Altered Microbiota in Cirrhosis and Its Relationship to the Development of Infection. Clin. Liver Dis..

[195] Sun Q., Dai H., Wang S., Chen Y., Shi H. (2023). Progress in research on the role played by myeloid-derived suppressor cells in liver diseases. Scand. J. Immunol..

[196] Zhou C.B., Zhou Y.L., Fang J.Y. (2021). Gut Microbiota in Cancer Immune Response and Immunotherapy. Trends Cancer.

[197] Tabrizian P., Jibara G., Shrager B., Schwartz M., Roayaie S. (2015). Recurrence of hepatocellular cancer after resection: Patterns, treatments, and prognosis. Ann. Surg..

[198] Lee D.D., Sapisochin G., Mehta N., Gorgen A., Musto K.R., Hajda H., Yao F.Y., Hodge D.O., Carter R.E., Harnois D.M. (2020). Surveillance for HCC After Liver Transplantation: Increased Monitoring May Yield Aggressive Treatment Options and Improved Postrecurrence Survival. Transplantation.

[199] Cong R., Ma X.H., Wang S., Feng B., Cai W., Chen Z.W., Zhao X.M. (2023). Application of ablative therapy for intrahepatic recurrent hepatocellular carcinoma following hepatectomy. World J. Gastrointest. Surg..

[200] Tassinari E., Rosellini M., Marchetti A., Mollica V., Massari F. (2024). What is the risk of hepatotoxicity induced by immune-checkpoint inhibitors and how can we avoid it?. Expert Opin. Drug Metab. Toxicol..

[201] Bjornsson E.S. (2021). Clinical management of patients with drug-induced liver injury (DILI). United Eur. Gastroenterol. J..

[202] Yun K.M., Bazhenova L. (2023). Management of toxicities associated with immune checkpoint inhibitors. Clin. Adv. Hematol. Oncol..

[203] Chen H., Yang C., Yan S., Liu X., Zhou L., Yuan X. (2024). Sarcopenia in cirrhosis: From pathophysiology to interventional therapy. Exp. Gerontol..

[204] Verheul H.M., Pinedo H.M. (2007). Possible molecular mechanisms involved in the toxicity of angiogenesis inhibition. Nat. Rev. Cancer.

[205] Watson N., Al-Samkari H. (2021). Thrombotic and bleeding risk of angiogenesis inhibitors in patients with and without malignancy. J. Thromb. Haemost..

[206] Jachs M., Reiberger T. (2021). Prevention of Variceal Bleeding and Rebleeding by Nonselective Beta-Blockers: A Tailored Approach. Clin. Liver Dis..

[207] Das A., Sil A., Khan I.A., Bandyopadhyay D. (2023). Dermatological adverse drug reactions to tyrosine kinase inhibitors: A narrative review. Clin. Exp. Dermatol..

[208] Lustberg M.B., Kuderer N.M., Desai A., Bergerot C., Lyman G.H. (2023). Mitigating long-term and delayed adverse events associated with cancer treatment: Implications for survivorship. Nat. Rev. Clin. Oncol..

[209] Meriggi F., Graffeo M. (2021). Clinical Characterisation and Management of the Main Treatment-Induced Toxicities in Patients with Hepatocellular Carcinoma and Cirrhosis. Cancers.

[210] Song Y.G., Yoo J.J., Kim S.G., Kim Y.S. (2024). Complications of immunotherapy in advanced hepatocellular carcinoma. J. Liver Cancer.

[211] Vogel A., Kelley R.K., Johnson P., Merle P., Yau T., Kudo M., Meyer T., Rimassa L. (2023). Predictive and Prognostic Potential of Liver Function Assessment in Patients with Advanced Hepatocellular Carcinoma: A Systematic Literature Review. Liver Cancer.

[212] Costa F., Wiedenmann B., Roderburg C., Mohr R., Abou-Alfa G.K. (2023). Systemic treatment in patients with Child-Pugh B liver dysfunction and advanced hepatocellular carcinoma. Cancer Med..

[213] Hanzel G.S., Dixon S., Goldstein J.A. (2017). Prioritizing and Combining Therapies for Heart Failure in the Era of Mechanical Support Devices. Interv. Cardiol. Clin..

[214] Palmer D.H., Malagari K., Kulik L.M. (2020). Role of locoregional therapies in the wake of systemic therapy. J. Hepatol..

[215] Agarwal P.D., Phillips P., Hillman L., Lucey M.R., Lee F., Mezrich J.D., Said A. (2017). Multidisciplinary Management of Hepatocellular Carcinoma Improves Access to Therapy and Patient Survival. J. Clin. Gastroenterol..

[216] Gewandter J.S., Dale W., Magnuson A., Pandya C., Heckler C.E., Lemelman T., Roussel B., Ifthikhar R., Dolan J., Noyes K. (2015). Associations between a patient-reported outcome (PRO) measure of sarcopenia and falls, functional status, and physical performance in older patients with cancer. J. Geriatr. Oncol..

[217] Benoist S., Brouquet A. (2015). Nutritional assessment and screening for malnutrition. J. Visc. Surg..

[218] Liu Y., Ji F., Nguyen M.H. (2023). Sarcopenia in cirrhosis: Epidemiology, diagnosis, management and prognosis. Curr. Opin. Gastroenterol..

[219] Di Maio M. (2025). Reading and Interpreting Quality-of-Life Results in Cancer Trials. NEJM Evid..

[220] Gaugain L., Cawston H., Dubois de Gennes C., Sanchez Alvares J., Nahon P., Mazaleyrat B., Le Dissez C. (2023). Cost-utility analysis of atezolizumab with bevacizumab in untreated unresectable or advanced hepatocellular carcinoma in France. PLoS ONE.

[221] Zhang C., Zhang C., Wang H. (2023). Immune-checkpoint inhibitor resistance in cancer treatment: Current progress and future directions. Cancer Lett..

[222] Ma C., Li Y., Li M., Lv C., Tian Y. (2025). Targeting immune checkpoints on myeloid cells: Current status and future directions. Cancer Immunol. Immunother..

[223] Wang L., Zhang L., Zhang Z., Wu P., Zhang Y., Chen X. (2024). Advances in targeting tumor microenvironment for immunotherapy. Front. Immunol..

[224] Rodriguez-Negrete E.V., Galvez-Martinez M., Sanchez-Reyes K., Fajardo-Felix C.F., Perez-Resendiz K.E., Madrigal-Santillan E.O., Morales-Gonzalez A., Morales-Gonzalez J.A. (2024). Liver Cirrhosis: The Immunocompromised State. J. Clin. Med..

[225] Solomon B.J., Beavis P.A., Darcy P.K. (2020). Promising Immuno-Oncology Options for the Future: Cellular Therapies and Personalized Cancer Vaccines. American Society of Clinical Oncology Educational Book.

[226] Rojas-Quintero J., Diaz M.P., Palmar J., Galan-Freyle N.J., Morillo V., Escalona D., Gonzalez-Torres H.J., Torres W., Navarro-Quiroz E., Rivera-Porras D. (2024). Car T Cells in Solid Tumors: Overcoming Obstacles. Int. J. Mol. Sci..

[227] Zhang L., Pakmehr S.A., Shahhosseini R., Hariri M., Fakhrioliaei A., Karkon Shayan F., Xiang W., Karkon Shayan S. (2023). Oncolytic viruses improve cancer immunotherapy by reprogramming solid tumor microenvironment. Med. Oncol..

[228] Zhang X.C., Zhou Y.W., Wei G.X., Luo Y.Q., Qiu M. (2024). Locoregional therapies combined with immune checkpoint inhibitors for liver metastases. Cancer Cell Int..

[229] Shin H.P., Lee M., Jeon J.W. (2025). Spectrum of therapeutic options in hepatocellular carcinoma. J. Exerc. Rehabil..

[230] Vo Quang E., Shimakawa Y., Nahon P. (2021). Epidemiological projections of viral-induced hepatocellular carcinoma in the perspective of WHO global hepatitis elimination. Liver Int..

[231] Siegel E.M., Ulrich C.M., Shibata D. (2023). Risk Stratification for Early-onset Colorectal Cancer Screening: Are We Ready for Implementation?. Cancer Prev. Res..

[232] Tang M., Pearson S.A., Simes R.J., Chua B.H. (2023). Harnessing Real-World Evidence to Advance Cancer Research. Curr. Oncol..

